# FFCD-1004 Clinical Trial: Impact of Cytidine Deaminase Activity on Clinical Outcome in Gemcitabine-Monotherapy Treated Patients

**DOI:** 10.1371/journal.pone.0135907

**Published:** 2015-08-26

**Authors:** Cindy Serdjebi, Johan Gagnière, Jérôme Desramé, Francine Fein, Rosine Guimbaud, Eric François, Thierry André, Jean-François Seitz, Carole Montérymard, Dominique Arsene, Julien Volet, Abakar Abakar-Mahamat, Thierry Lecomte, Véronique Guerin-Meyer, Jean-Louis Legoux, Gaël Deplanque, Pierre Guillet, Joseph Ciccolini, Côme Lepage, Laetitia Dahan

**Affiliations:** 1 SMARTc, CRO2 INSERM UMR S_911, Marseille, France; 2 University Hospital of Clermont Ferrand, Clermont Ferrand, France; 3 Center Jean Mermoz, Lyon, France; 4 University Hospital of Besançon, Besançon, France; 5 University Hospital of Toulouse, Toulouse, France; 6 Center Antoine Lacassagne, Nice, France; 7 University Hospital of Saint-Antoine and Pierre et Marie Curie, Assistance Publique des Hôpitaux de Paris, Paris, France; 8 University Hospital of La Timone, Assistance Publique des Hôpitaux de Marseille, Marseille, France; 9 Fédération Francophone de Cancérologie Digestive FFCD, Dijon, France; 10 University Hospital of Caen, Caen, France; 11 University Hospital of Reims, Reims, France; 12 University Hospital of Nice, Nice, France; 13 University Hospital of Tours, Tours, France; 14 University Hospital of Angers, Angers, France; 15 Regional Hospital of Orleans, Orleans, France; 16 St Joseph Hospital, Paris, France; 17 Hospital of Toulon, Toulon, France; 18 University Hospital of Dijon, Dijon, France; Cardiff University, UNITED KINGDOM

## Abstract

**Purpose:**

Because cytidine deaminase (CDA) is the key enzyme in gemcitabine metabolism, numerous studies have attempted to investigate impact of CDA status (i.e. genotype or phenotype) on clinical outcome. To date, data are still controversial because none of these studies has fully investigated genotype-phenotype CDA status, pharmacokinetics and clinical outcome relationships in gemcitabine-treated patients. Besides, most patients were treated with gemcitabine associated with other drugs, thus adding a confounding factor. We performed a multicenter prospective clinical trial in gemcitabine-treated patients which aimed at investigating the link between CDA deficiency on the occurrence of severe toxicities and on pharmacokinetics, and studying CDA genotype-phenotype relationships.

**Experimental design:**

One hundred twenty patients with resected pancreatic adenocarcinoma eligible for adjuvant gemcitabine monotherapy were enrolled in this study promoted and managed by the Fédération Francophone de Cancérologie Digestive. Toxicities were graded according to National Cancer Institute’s Common Terminology Criteria for Adverse Events Version 4. They were considered severe for grade ≥ 3, and early when occurring during the first eight weeks of treatment. CDA status was evaluated using a double approach: genotyping for 79A>C and functional testing. Therapeutic drug monitoring of gemcitabine and its metabolite were performed on the first course of gemcitabine.

**Results:**

Five patients out of 120 (i.e., 4.6%) were found to be CDA deficient (i.e., CDA activity <1.3 U/mg), and only one among them experienced early severe hematological toxicity. There was no statistically significant difference in CDA activity between patients experiencing hematological severe toxicities (28.44%) and patients who tolerated the treatment (71.56%). CDA genetic analysis failed in evidencing an impact in terms of toxicities or in CDA activity. Regarding pharmacokinetics, a wide inter-individual variability has been observed in patients.

**Conclusion:**

This study, which included only 4.6% of CDA-deficient patients, failed in identifying CDA status as a predictive marker of toxicities with gemcitabine. A lack of statistical power because of smoothing effect of CDA variability as compared with real life conditions could explain this absence of impact.

**Trial Registration:**

ClinicalTrials.gov NCT01416662

## Introduction

Gemcitabine is an antimetabolite drug widely prescribed to treat various types of cancer, from solid tumors to hematological disorders such as pancreatic adenocarcinoma, non-small cell lung cancer, sarcoma or lymphoma, alone or in combination with others cytotoxics such as capecitabine, nab-paclitaxel or oxaliplatin [1–5]]. Since identification of cytidine deaminase (CDA) as the key enzyme involved in the metabolic pathway of gemcitabine in the early 90’s [6], many studies have been conducted to try to better understand whether changes in CDA activity could impact on efficacy and toxicity of gemcitabine-based regimen [7] The existing wide inter-individual variability in CDA activity allows patients to be sorted into 3 categories: poor metabolizers (PM), defined with a reduced CDA activity (CDA<1.3 U/mg), ultra-deaminators (UM), individuals with an increased CDA activity (>6 U/mg) and patients with a normal CDA phenotype (extensive metabolizers, EM) showing a median activity of 3.2 in adults and 3.4 U/mg in children [8].

This marked variability among activities is partly linked to genetic and epigenetic polymorphisms [9]. *CDA* is indeed a highly polymorphic gene [10], with synonymous variations in intron with little consequence on the phenotype to non-synonymous non-sense polymorphisms leading to a truncated protein [11]. Some of these variations have been well studied with respect to clinical outcome upon gemcitabine intake. Among them, the non-synonymous rs2072671 encodes for a substitution of a lysine into a glutamine because of a replacement of the adenine by a cytosine in position 79 in the coding sequence. According to Micozzi et al. [12], this modification in the amino-acid sequence does not impact on the catalytic event. Many studies have been performed to establish clear correlation between this polymorphism and CDA phenotype [7]. To date, Giovannetti et al. and Baker et al. correlated the wild type genotype (*A/A* encoding for Lys27Lys enzyme) with a lower mean CDA activity [11, 13, 14] while our team did not clearly evidence a link between CDA genotype and functionality with respect to cytidine deamination [8]. Very recently, the impact of *79A>C* polymorphism on the response and hematological toxicities in gemcitabine-treated patients has been studied in a meta-analysis, encapsulating 6 studies performed in 2013 [15]. This meta-analysis suggested that the *A/A* genotype seems to be a protective factor with respect to incidence of severe anemia, whereas the *C/C* genotype did not show a significant impact on the response rate in non-small cell lung cancer (NSCLC) patients treated with gemcitabine.

A decrease in CDA activity leads to a decrease of gemcitabine clearance. We already evidenced an overexposure to gemcitabine due to CDA deficiency in mice treated with gemcitabine [8]. Many published studies in human have confirmed these results, reporting a decrease in gemcitabine clearance correlated with either a given *CDA* genotype [15, 16] or reduced deamination function [7, 8, 17]. Conversely, we have shown that UM pancreatic cancer patients (i.e., CDA activity > 6 U/mg) were 5-times more likely to have progressive disease upon gemcitabine intake, as compared with patients with normal CDA status [18]. Despite the lack of pharmacokinetic support in this study, this may be explained by an over-elimination of gemcitabine by the CDA hyperactivity, thus resulting in lack of efficacy.

To date, none of these studies fully investigated genotype-phenotype-pharmacokinetics and clinical outcome relationships with gemcitabine. Such a study would highlight how genetic events could influence the functional activity of CDA, thus confirming that CDA status is a predictive factor of a decrease in gemcitabine clearance, with consequently a higher incidence of severe toxicity. Here, we performed a prospective clinical trial in gemcitabine-treated patients with resected pancreatic cancer which aimed at investigating the impact of CDA deficiency on the occurrence of toxicities and on pharmacokinetics, but also to better understand CDA genotype-phenotype relationships. This study was promoted and managed by the Fédération Francophone de Cancérologie Digestive (FFCD).

## Patients & Methods

### Objectives

The main objective of this trial was to evaluate the ability of CDA to predict the occurrence of early severe hematological toxicity upon gemcitabine. The secondary objectives were to evaluate the capability of CDA to predict the occurrence of early severe non hematological toxicities, and the hematological toxicities induced by gemcitabine overall the treatment, to evaluate the impact of CDA activity on pharmacokinetics of gemcitabine and its metabolite, and to study CDA genotype-phenotype relationships. An exploratory analysis was planned to study if CDA could be a prognostic factor and a marker for gemcitabine efficacy.

### Patients and treatment

**Fig 1:**
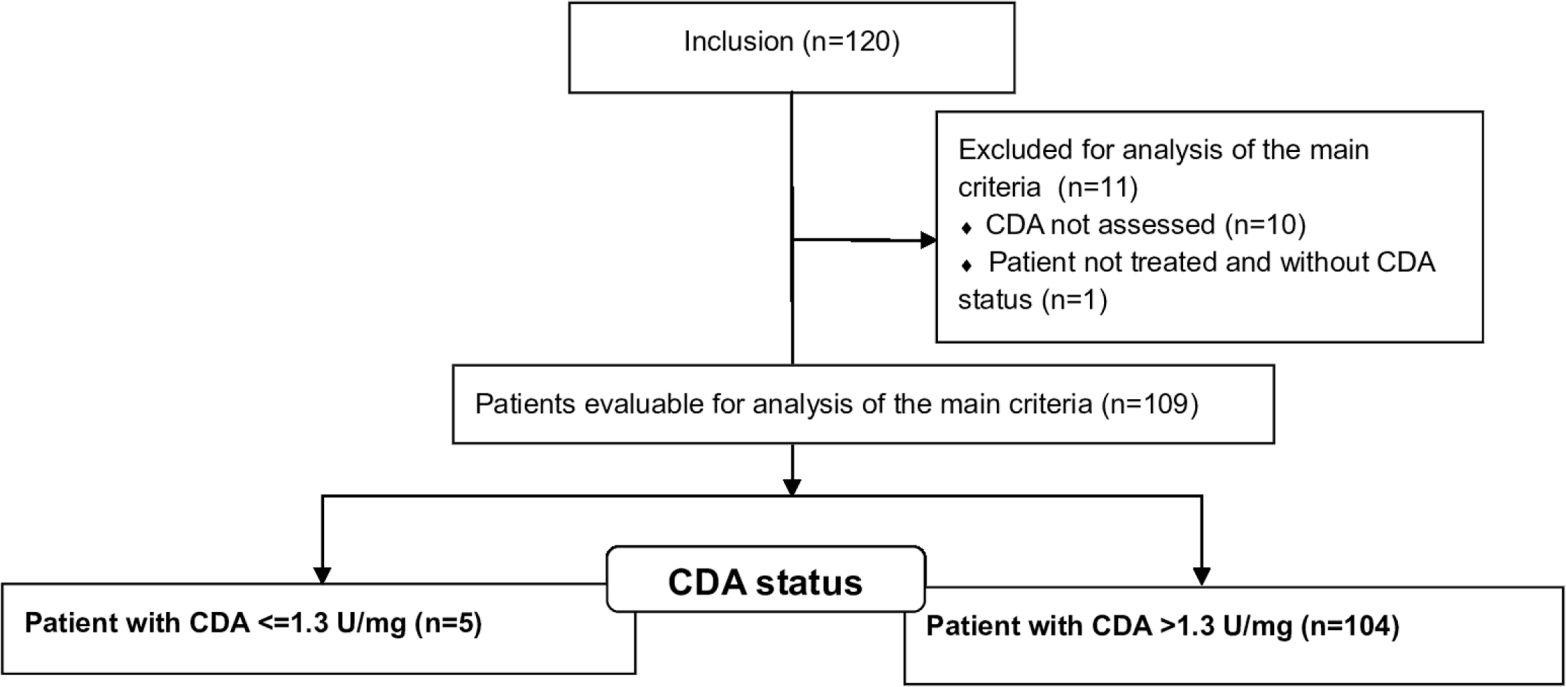
Consort flowchart of the primary objective in the FFCD-1004 clinical trial.

Adult patients with resected pancreatic adenocarcinoma (R0: resection for cure or R1: microscopic residual tumor) were included from 31 French clinical centers in this study. Main non-inclusion criteria were metastatic or locally advanced disease, presence of infectious syndrome, and previous chemotherapy (within the ten years before inclusion). All patients were eligible for adjuvant gemcitabine-monotherapy as follows: 30-minutes IV-infusion of 1000 mg/m^2^ every week for 3 consecutive weeks followed by one week off for 6 courses. Demographic (e.g age, sex weight, body surface area), clinical (e.g. tumor size, localization, and tumor classification using TNM, disease grade, World Health Organization (WHO) status, resection margin) and biological data (e.g. data about liver, renal and cardiac functions, whole blood count) were collected prior and during treatment. Toxicities were graded according to the National Cancer Institute’s Common Terminology Criteria for Adverse Events Version 4. Toxicities were considered as severe for grade ≥ 3, and early when they occurred during the first eight weeks of treatment. Written informed consent was provided prior enrolment in accordance with the Declaration of Helsinki and this study was approved by local ethical committee (Comité de Protection des Personnes Sud Méditerranée 1) in January 2011. The authors confirmed that all ongoing and related trials for this drug are registered: trial was registered at the ANSM as 2010-022987-11 and on the clinicaltrial.gov website in June 2011. The patients were recruited between July 2011 and June 2013 with a 2-years follow-up (**Fig 1, S1 Checklist and S1 Protocol**).

### Determination of CDA status

CDA status was evaluated using a double approach: a phenotypic assay and a genetic analysis. Blood samples (one dry tube and one EDTA tube for evaluation of CDA activity and genotyping test, respectively) were collected from patients prior to treatment. Seric fraction was isolated after centrifugation of the dry tube, whereas lymphocyte DNA was extracted from the EDTA tube using EZ1 DNA Blood Kit (Qiagen). DNA and seric fraction was then conserved at -20 and -80°C respectively until analysis. CDA activity was evaluated using a previously developed method, already used in a routine clinical practice in our hospital [8]. Briefly, as CDA is physiologically responsible for the deamination of cytidine by releasing ammonium, patients serum were incubated overnight ex vivo with cytidine and the quantity of released ammonium was measured by visible spectrophotometry. Seric proteins amount was also assayed using Pierce method as CDA activity is finally expressed with respect to the seric proteins amount. Patients were considered as PM when their CDA activity was ≤1.3 U/mg, a threshold previously determined from our retrospective study [8]. As *CDA* rs2072671 SNP (i.e. 79A>C) is the most common non-synonymous exonic variant found in the Caucasian population [12], we screened for this genetic polymorphism in the cohort, using High Resolution Melting (HRM) analysis on LightCycler480. After amplification of each patient DNA in duplicate, the melting curve (from 70° to 95°C) was compared to standards in order to establish the genotype.

### Gemcitabine and dFdU therapeutic drug monitoring (TDM)

A pharmacokinetic study was performed. It consisted in sampling patients during the first round of gemcitabine administration to evaluate circulating plasma levels of both gemcitabine and its inactive metabolite dFdU. Blood samples were collected before starting the infusion, at the end and 30 and 90 minutes after the end of infusion in EDTA tube containing tetrahydrouridine (THU) to prevent *in silico* deamination by CDA. Plasma fraction was immediately isolated after centrifugation and aliquots were stocked at -80°C until analysis. Gemcitabine and dFdU were simultaneously measured using LC-MSMS assay, adapted from by Zhou et al. [19], and validated according to European Medicines Agency (EMA) and Food and Drug Administration (FDA) guidelines. Briefly, 50 μl of drug-free plasma and 500 μl of precipitating solution containing acetonitrile and internal standard (abacavir ABC) are added to a 50-μl test sample. After vortex-mixing for 90 sec, samples were centrifuged during 10 minutes at 14 000 rpm at room temperature. The supernatant was then dry evaporated under a gentle stream of nitrogen at 45°C and the residue was dissolved in 600 μl of reconstitution solvent (5 mM ammonium acetate + 0.1% formic acid) by vortex-mixing for 15 sec. Then, samples were transferred into 96-well plate before analysis.

Chromatographic separations of gemcitabine, dFdU and ABC were performed on an UPLC system (Waters UPLC-TQD), using an Acquity UPLC HSS T3 column (1.8 μm x 2.1 x 100mm, Waters) maintained at 40°C. The mobile phase was composed of an acetate buffer (5 mM ammonium acetate, 0.1%, formic acid) as aqueous phase and an organic phase composed of acetonitrile. A 5 μl-volume sample was injected and eluted with a gradient of aqueous and organic phases at a constant flow rate of 0.250 ml/min for a total run time of 6 minutes. A Waters tandem quadrupole detection system with an electrospray ionization (ESI) source operating in positive mode for gemcitabine and ABC and negative mode for dFdU was used to obtain mass and product ion spectra (MS and MS-MS). MassLynx software version 4.1 was used for data acquisition and processing. Mass transitions of *m/z* 264.05 -> 112.05, 287.10 -> 191.10 were optimized for gemcitabine and ABC, respectively. For dFdU, two transitions have been used: 263.05 -> 111.05 for quantification and 263.05 -> 219.95 for qualification.

The resulting pharmacokinetics data were analyzed using pharmacokinetics software KineticPro (WGroup, France). Bayesian estimation was used to estimate individual pharmacokinetics parameters and to simulate plasma exposure of patients, based on previously published pharmacokinetic parameters [20].

### Statistical analysis

The main objective of the trial was to evaluate the capability of CDA to predict the occurrence of early severe hematological toxicity upon gemcitabine. In order to bring out a positive likelihood ratio of CDA more than 3.5 (H_1_), objective reached when sensitivity is more than 70% and specificity is more than 80% with an α risk of 5% and a power of 80%, 107 patients had to be included. With an estimated 10% of lost-to-follow-up patients or patients not treated, 120 patients were finally included. The probability of severe hematological toxicity without CDA knowledge was estimated at 12.5%, based on retrospective published data [8]. Primary criterion, safety secondary endpoints (i.e., overall toxicities and non-hematological ones) and exploratory analyses were done on the set of patients receiving at least one dose of gemcitabine and with a CDA fully evaluable status. The primary criterion was measured with positive and negative likelihood ratios, sensitivity and specificity, and the positive and negative predictive values (with their 95% confidence intervals). Time to event endpoints (e.g time to grade 3–4 hematological (or non-hematological) toxicity appearance as-well-as overall survival) were estimated using Kaplan-Meier method. A series of clinical parameters measured at the inclusion were evaluated as prognostic factors on the overall survival in a univariate and a multivariate analyses. We also tested the influence of 79A>C genotype on incidence of severe toxicities using Chi^2^-test. In this aim, we gathered the heterozygous and homozygous mutated patients as “mutated” patients’ status.

## Results

### Patients and clinical outcome

One hundred and twenty patients have been included in this prospective study, from 31 different French centers, with a mean age of 65.8 years (sd 10.05) and a sex ratio of 1.61 (**Table 1**). One hundred and nineteen patients (99.9%) have received at least one dose of gemcitabine.

**Table 1:** Patients’ characteristics.

Clinical characteristics	Clinical characteristics subgroups	Number of patients (%) N = 120
Sex	male	74 (61.7%)
	female	46 (38.3%)
Age	median	66.72
	minimum	30.6
	maximum	84.9
WHO performance status	0	46 (38.3%)
	1	68 (56.7%)
	2	6 (5.0%)
Tumor localization	head	96 (80.0%)
	body	8 (6.7%)
	tail	11 (9.2%)
	head and body	1 (0.8%)
	head and tail	1 (0.8%)
	body and tail	3 (2.5%)
Disease grade (TNM classification)	T1/T2	24 (20.0%)
	T3/T4	96 (80.0%)
	N+	84 (70.0%)
Resection margin	R0	106 (88.3%)
	R1	14 (11.7%)

The median survival was 29.08 months [95% CI, 25.1 to NA], with 6-months and 12-months survival rates of 95.8 and 79.82%, respectively. In terms of toxicity, 25 (23%) patients declared early severe hematological toxicities, 20 (18%) displayed other early severe toxicities. Maximal gemcitabine caused-toxicities during treatment are summarized in **Table 2**.

**Table 2:** Maximal gemcitabine-caused toxicities over treatment.

Types of toxicity	Subtypes of toxicity	No. of patients (%)	
Toxicity grades		Grades 1–2	Grades 3–4
Hematological	Anemia	110 (92.4%)	5 (4.2%)
	Leucopenia	81 (68.1%)	9 (7.6%)
	Neutropenia	64 (53.8%)	30 (25.2%)
	Thrombopenia	62 (52.1%)	1 (0.8%)
Non hematological	Nausea	65 (54.6%)	1 (0.8%)
	Vomiting	33 (27.7%)	0 (0%)
	Diarrhea	66 (55.5%)	1 (0.8%)
	Asthenia	90 (75.6%)	3 (2.5%)

### CDA status

One hundred and nine and ninety-two patients were analyzable for CDA phenotyping and genotyping tests, respectively. Five patients out of 109 (i.e. 4.6%) were found to be PM and one patient (i.e. 0.9%) displayed CDA activity greater than 6 U/mg (UM). Among the CDA-deficient patients, only one experienced early severe hematological toxicities. Median CDA activity was not significantly different between patients who had early severe toxicities and patients with no such toxicities (2.35 vs 2.25 respectively p>0.05, **Table 3**). In the same manner, with respect to severe hematological and non-hematological toxicities all over the treatment, CDA activity was also similar when comparing patients with toxicities and patients with no toxicities. Age and disease stage at the diagnostic were both correlated with overall survival in univariate and multivariate analysis (**Table 4**), whereas no influence of CDA activity could be evidenced neither on the overall survival (p = 0.28 in univariate analysis).

**Table 3:** Toxicities during treatment according to CDA activity (n = 109).

	No. of patients with severe hematological toxicities in the first eight weeks of treatment (%)		
	no	yes	p-value
n	84	25	
CDA median activity	2.25	2.35	p = 0.87
Q1-Q3	1.80–2.80	1.89–3.32	
CDA ≤ 1.3 U/mg *vs*. > 1.3 U/mg	4 (4.76%) *vs*. 80 (95.24%)	1 (4%) *vs*. 24 (96%)	p = 0.99
Total	84 (100%)	25 (100%)	109 (100%)
	No. of patients with severe hematological toxicities all over the treatment (%)		
	no	yes	p-value
CDA median activity	2.25	2.35	p = 0.67
Q1-Q3	1.78–2.82	1.89–3.11	
CDA ≤ 1.3 U/mg *vs*. > 1.3 U/mg	4 (5.13%) *vs*. 74 (94.87%)	1 (3.23%) *vs*. 30 (96.77%)	p = 0.99
Total	78 (100%)	31 (100)	109 (100%)
	No. of patients with severe non hematological toxicities all over the treatment (%)		
	no	yes	p-value
CDA median activity	2.215	2.3	p = 0.15
Q1-Q3	1.73–3.02	1.99–2.82	
CDA ≤ 1.3 U/mg *vs*. > 1.3 U/mg	5 (6.41%) *vs*. 73 (93.6%)	0 (0%) *vs*. 31 (100%)	p = 0.32
Total	78 (100%)	31 (100%)	109 (100%)

**Table 4:** Median overall survival according to different criteria.

Clinical criteria	Median overall survival (months)			95% CI
Age	<65 y.o *vs*. *≥65 y*.*o*	34.4 *vs*. 26.5	p = 0.0253	[25.7-NA] *vs*. [20.9–31.3]
Disease stage	T1-T2 *vs*.T3-T4	31.3 *vs*. 29	p = 0.0408	[25.1—NA] *vs*. [20.9—NA]

Studying CDA genotype showed that 59.8% of patients displayed the 79A>C polymorphism. No correlation was evidenced between this CDA genotype and occurrence of toxicities, whether hematological or not, and CDA activity was similar in mutated patients group and the non-mutated patients group (p = 0.61).

### Pharmacokinetic study

**Fig 2:**
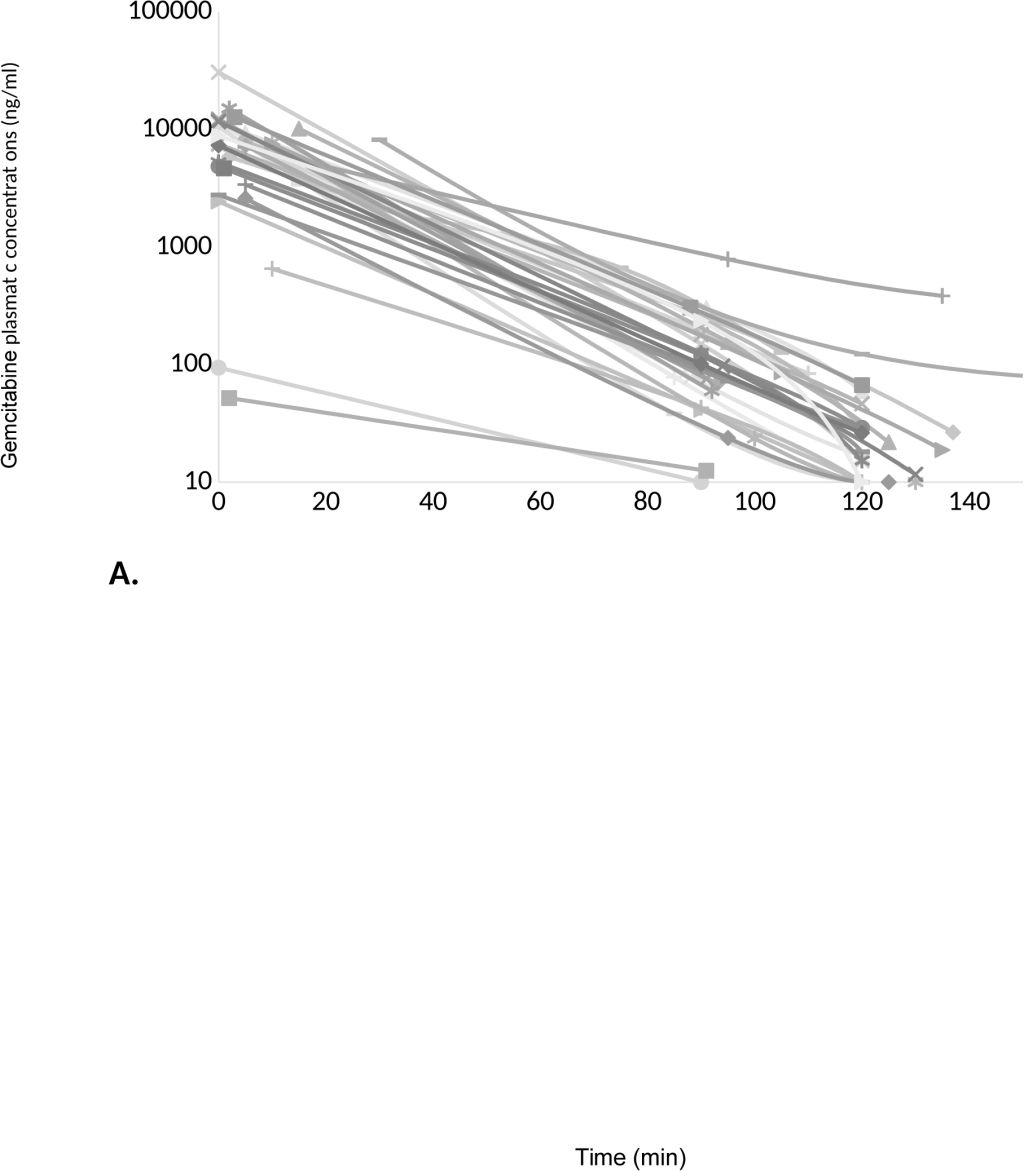
Pharmacokinetics profiles of gemcitabine (A) and its metabolite (B) overtime during the first round of gemcitabine administration in pancreatic adenocarcinoma cancer patients. Patients have been sampled before infusion, at the end, and 90 and 120 minutes after ending the infusion. A wide inter-individual variability is observed, either for gemcitabine or for dFdU.

A total of 39 out of the 120 patients (33%) initially involved in the clinical trial were enrolled in the additional pharmacokinetic study (Fig 2). Administration protocol was not strictly respected for 9 patients (infusion time superior to 45 minutes) and kinetics were not complete for 2 patients. Among patients with a carefully-driven administration protocol, gemcitabine plasma concentrations ranged from 19.8 to 2.77 μg/ml at end of infusion and AUC from 1.23x10^5^ to 4.80x10^8^ ng/ml*min, thus evidencing a wide inter-individual variability in gemcitabine exposure in patients.

## Discussion

**Fig 3:**
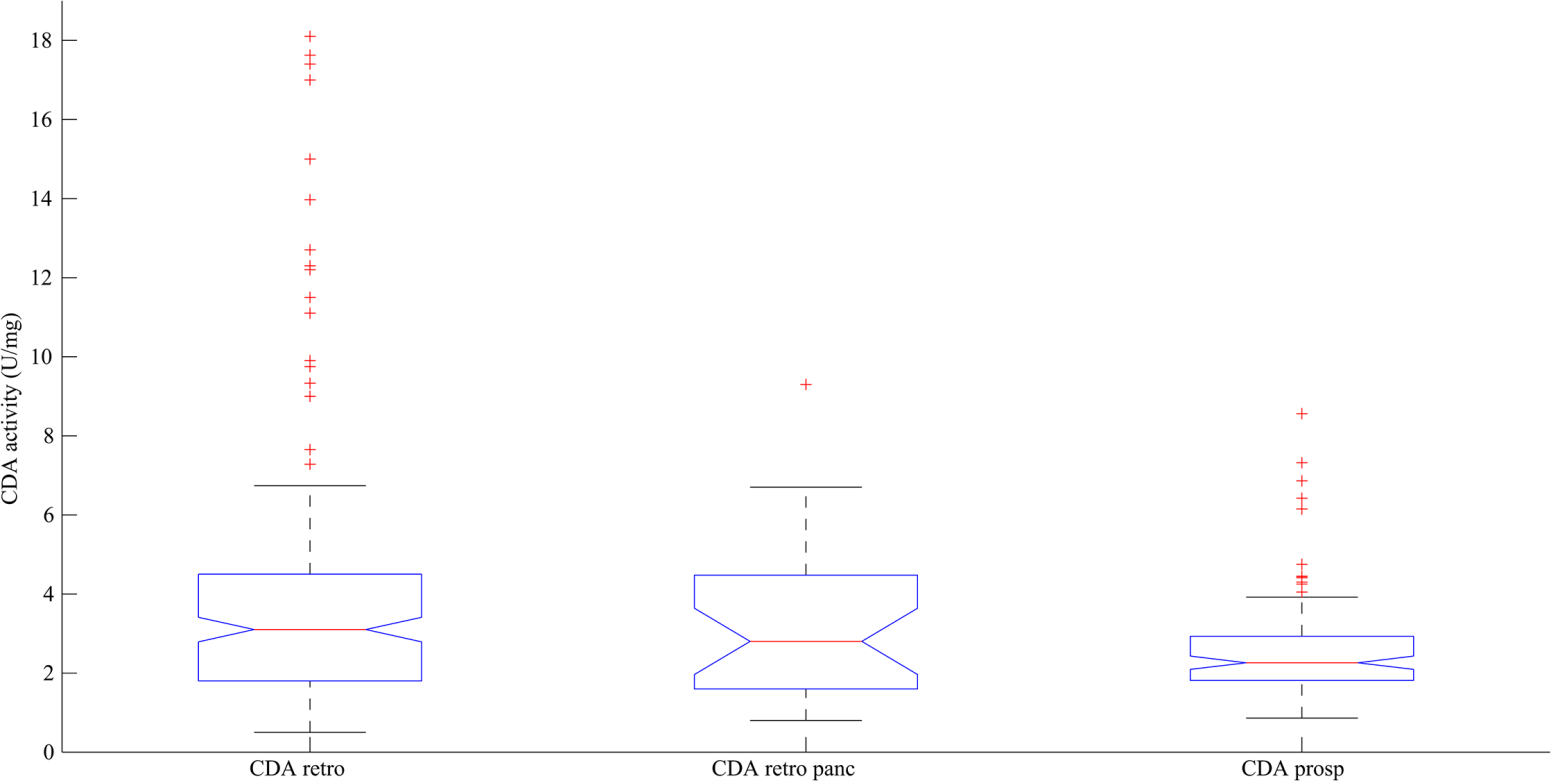
Boxplots of CDA activity in prospective compared to retrospective study. CDAretro stands for CDA activity in gemcitabine-treated patients for various types of cancers in the retrospective study. The boxplot in the middle represents CDA activity in the subgroup of gemcitabine-monotherapy patients in the retrospective study. CDA activities of patients from the prospective study are represented in the boxplot on the right. Isolating gemcitabine-monotherapy treated patients leads to a smoothing effect in CDA activity variability.

Developing and implementing new tests in a routine clinical practice allowing to stratify patients according to their characteristics has become a rising trend. To date, many pharmacogenomic tests are available to ensure a better response with respect to the treatment in patients. Additionally, some pharmacogenetic assays are recommended to be performed prior to the start of treatment, such as TMPT for azathioprine or 6-mercaptopurine [21]. Regarding to CDA, since evidence has been made that it is the key enzyme involved in gemcitabine deactivation, many studies focused on CDA status and toxicities or pharmacokinetics relationships. All studies are consistent with the fact that an impairment in CDA functionality leads to gemcitabine overexposure caused by a decrease in gemcitabine liver clearance. However, with respect to the *79A>C* SNP, available data are not consistent, when not conflictual [14, 22–24]. The discrepancies in CDA pharmacogenetic studies partly explain why there is still no recommendation for testing CDA at bedside before initiating gemcitabine-treatment. Thus, ensuring a better tolerance of gemcitabine and a better management of toxic risk in patients treated with gemcitabine depends only on the goodwill on the medical team, and on the availability of a surrogate CDA test in the institute. Here, we proposed a prospective study to confirm whether CDA status could be a predictive factor of toxicities in patients under gemcitabine-based treatment. The originality of this study was to address the issue of genotype, phenotype, pharmacokinetics and clinical outcome. The study confirmed the wide inter-individual variability in pharmacokinetics, already described elsewhere [8, 25]. Regarding to CDA, unfortunately, no association between *CDA* genotype nor CDA phenotype and toxicity was evidenced here. This absence of significant association between CDA and toxicity could be explained by the surprisingly weak proportion of CDA-deficient patients in this study. Indeed, the design of this study was inspired from a previous one [8], involving unselected patients treated with gemcitabine alone or in combination for various types of cancers. In this previous retrospective study involving routine patients of our institute, 10% of individuals were PM according to CDA and we showed that patients with decreased CDA activity were significantly more at risk to experience severe toxicities. However, this was not confirmed in the subgroup of pancreatic cancer patients treated with gemcitabine only, suggesting a lack of statistical power. In this new study, variability of CDA was found to be considerably reduced when compared to the first trial, but comparable to the retrospective pancreatic patients subgroup (Fig 3). Only 4.59% of patients displayed CDA deficiency, thus preventing to perform any statistical test. This low incidence of CDA deficiency may be explained by a smoothing effect due to performing a clinical trial with overselected patients. Similarly, only 0.9% of patients displayed UM phenotype, while 14% were found in the previous study [18]. Here, drastic inclusion criteria used to meet the requirements of a prospective trial apparently had a negative impact on CDA natural variability.

To summarize, this prospective study failed in identifying CDA as a predictive marker of toxicities under gemcitabine treatment. A lack of statistical power because of smoothing effect of CDA variability as compared with real life conditions could explain this.

## Supporting Information

S1 Checklist TREND statement checklist. (PDF)

S1 Protocol Protocol of the FFCD-1004 clinical trial. (PDF)
