# Supplementary material for: FFCD-1004 Clinical Trial: Impact of Cytidine Deaminase Activity on Clinical Outcome in Gemcitabine-Monotherapy Treated Patients
Source: PLoS One. 2015 Aug 26;10(8):e0135907. doi: 10.1371/journal.pone.0135907 (PMC4550302; doi:10.1371/journal.pone.0135907)
Supplement: S1 Protocol — (PDF) [file pone.0135907.s002.pdf]

PHARMACOGENETIQUE DE LA GEMCITABINE : ETUDE DE L'IMPACT DU POLYMORPHISME GENETIQUE DE LA CYTIDINE DEAMINASE (CDA) SUR LA TOXICITE DANS LES ADENOCARCINOMES PANCREATIQUES RESEQUES

ESSAI FFCD 1004 - PRODIGE - GERCOR

EudraCT n° : 2010-022987-11

COORDONNATEUR

Dr DAHAN Laetitia
Service d'Oncologie Digestive, CHU TIMONE Adulte
Pôle Oncologie, Spécialités Médicales et Chirurgicales
264 rue St Pierre
13385 Marseille Cedex 05
Tél : 04 91 38 60 23 - Fax : 04 91 38 48 73, courriel : laetitia.dahan@mail.ap-hm.fr

RESPONSABLE DE L'ETUDE BIOLOGIQUE

Dr Joseph CICCOLINI
Laboratoire de transfert en Oncologie Biologique APHM et Laboratoire de Pharmacocinétique et Toxicologie
CHU Timone
264 rue St Pierre
13385 Marseille 05
Tél : 04 91 69 88 82/83- Fax : 04 91 69 01 71, courriel : joseph.ciccolini@univmed.fr

COMITE DE REDACTION

F. Bonnetain, J. Ciccolini, T. Conroy, L. Dahan, P. Hammel, E. Mitry, JF. Seitz, F. Subtil

PROMOTEUR ET CENTRE DE RANDOMISATION -GESTION-ANALYSE (CRGA)

Fédération Francophone de Cancérologie Digestive (FFCD) :

Directrice Administrative : **Cécile GIRAULT**
Faculté de Médecine, 7 Boulevard Jeanne d'Arc, BP 87900
21079 Dijon Cedex, France
Tél: 03 80 66 80 13 - Fax: 03 80 38 18 41
E-mail : cgirault@u-bourgogne.fr

Méthodologiste : **Fabien SUBTIL**
Faculté de Médecine, 7 Boulevard Jeanne d'Arc, BP 87900
21079 Dijon Cedex, France
Tél : 03 80 39 34 71- Fax: 03 80 38 18 41
E-mail: fabien.subtil@u-bourgogne.fr

Chef de projets: **Martina SCHNEIDER**
Faculté de Médecine, 7 Boulevard Jeanne d'Arc, BP 87900
21079 Dijon Cedex, France
Tél : 03 80 39 34 83 - Fax : 03 80 38 18 41
E-mail : martina.schneider@u-bourgogne.fr

SOMMAIRE

ACCORD PROTOCOLAIRE ET BPC 3

CONTACTS FFCD 4

SYNOPSIS 5

1. INTRODUCTION ET JUSTIFICATION DE L'ETUDE 8

2. DONNEES DE LA LITTERATURE ET PRE-REQUIS 9

3. OBJECTIFS DE L'ETUDE 10

4. CRITERES DE SELECTION 11

5. BILAN INITIAL 12

6. INCLUSION 13

7. PRELEVEMENTS SANGUINS POUR LES ETUDES PHARMACOGENETIQUES ET PHARMACOCINETIQUES 13

8. TRAITEMENT PAR GEMCITABINE – ADAPTATION DES DOSES 15

9. EVALUATION DE LA SECURITE 16

10. SUIVI DES PATIENTS 18

11. CRITERES DE JUGEMENT 19

12. NOMBRE DE PATIENTS NECESSAIRES 20

13. GESTION DES DONNEES ET STATISTIQUES 21

14. COMITES 23

15. ASPECTS LEGAUX ET ETHIQUES 24

16. REGLES DE PUBLICATIONS 25

17. BIBLIOGRAPHIE 26

ANNEXE 1 : FICHE PRODUIT DU GEMZAR® 27

ANNEXE 2 : NOTE D'INFORMATION ET DE CONSENTEMENT 34

ANNEXE 3 : CRITERES DE TOXICITE NCI-CTC VERSION 4.0 38

ANNEXE 4 : FICHES DE PRELEVEMENT 39

ANNEXE 5 : REGLES DE PUBLICATION FFCD 41

ANNEXE 6: CERTIFICAT D'ASSURANCE 42

ANNEXE 7: AVIS DU CPP SUD MEDITERRANEE I 43

ANNEXE 8 : AUTORISATION DE L'AFSSAPS 45

ACCORD PROTOCOLAIRE ET BPC

FFCD 1004 : PHARMACOGENETIQUE DE LA GEMCITABINE : ETUDE DE L'IMPACT DU POLYMORPHISME GENETIQUE DE LA CYTIDINE DEAMINASE (CDA) SUR LA TOXICITE DANS LES ADENOCARCINOMES PANCREATIQUES RESEQUES

Numéro EudraCT : 2010-022987-11

Version 1.2 du 14/04/2011

Cette version du protocole est approuvée par:

Le Promoteur: Mme Cécile GIRAULT Date : 14/04/2011 Signature :

Le Coordonnateur: Dr Laëtitia DAHAN Date : 14/04/2011 Signature :

Je soussigné(e), Docteur

Après avoir lu le protocole, certifie m'engager à mener cet essai en respectant toutes ses directives, celles de la loi Huriet, de la Déclaration d'Helsinki et des Bonnes Pratiques Cliniques.

Je m'engage notamment à:

- faire signer à chaque patient un consentement écrit après lui avoir fait prendre connaissance de la note d'information qui lui est destinée,
- déclarer les événements indésirables graves dans les 24 heures après en avoir eu connaissance,
- respecter les critères d'inclusion et de non inclusion, ainsi que les dates de début et de fin d'étude,
- participer à la partie translationnelle de l'étude sous réserve de la signature par le patient du consentement spécifique et adresser les prélèvements selon les recommandations,
- remplir tous les items du cahier d'observation,
- répondre par téléphone ou par courrier aux demandes de corrections ou précisions concernant le cahier d'observation,
- accepter la visite d'un Attaché de Recherche Clinique (ARC) pour le monitorage et mettre à sa disposition le dossier médical des patients inclus dans l'étude, ainsi que les personnes mandatées par le promoteur ou les autorités de Santé pour la réalisation d'audit,
- accorder le temps nécessaire à l'ARC FFCD pour la signature des fiches, les réponses aux questions éventuelles et les actions à entreprendre,
- archiver et garder les documents de l'essai pendant 15 ans

Date :   Signature :

CACHET du CENTRE :

Envoyer l'original au CRGA de la FFCD – 7 bd Jeanne d'Arc – BP 87900 – 21079 Dijon Cedex

CONTACTS FFCD

NOM	FONCTION	TELEPHONE	TELECOPIE
**DIRECTION ADMINISTRATIVE**			
**Cécile GIRAULT**	Directrice Administrative	03 80 39 33 87	03 80 38 18 41
**Martina SCHNEIDER**	Directrice Administrative Adjointe	03 80 39 34 83	03 80 38 18 41
**Aurélie DUBREU**	Secrétariat de direction	03 80 38 13 14	03 80 38 18 41
**CENTRE DE RANDOMISATION - GESTION - ANALYSE**			
**Fabien SUBTIL**	Statisticien	03 80 39 34 71	03 80 38 18 41
**Emilie MAILLARD**	Statisticienne	03 80 39 34 81	03 80 38 18 41
**Jacques RICARD**	Informaticien	03 80 39 34 71	03 80 38 18 41
**Marie MOREAU**	Chef de Projets	03 80 39 34 04	03 80 38 18 41
**Géraldine VAUDRIT**	Assistante Chef de Projets	03 80 38 33 86	03 80 38 18 41
**Martina SCHNEIDER**	Chef de Projets	03 80 39 34 83	03 80 38 18 41
**Jaïque CARIO**	Assistante Chef de Projets	03 80 39 34 86	03 80 38 18 41
**Caroline CHOINE-POURRET**	ARC Coordonnateur	04 74 39 11 59	04 74 39 11 59
**Hicham FATTOUH**	ARC	04 78 66 13 08	04 78 66 13 08
**Florence GUILIANI-KPODOH**	ARC	09 71 26 83 54	09 71 26 83 54
**Armelle ISSA**	ARC	01 71 16 41 40	03 80 38 18 41
**Gaëlle LE PESSEC**	ARC	01 34 09 08 58	03 80 38 18 41
**Nicolas LE PROVOST**	ARC	09 63 51 97 83	05 56 05 03 59
**Fadil MASSKOURI**	Data Manager	03 80 39 34 05	03 80 38 18 41
**Charles FUCHEY**	Data Manager	03 80 39 34 81	03 80 38 18 41
**Floriane RICARD**	Data Manager	03 80 39 34 84	03 80 38 18 41

Vous pouvez également contacter le CRGA de la FFCD au : 03 80 66 80 13
(du lundi au vendredi de 8H00 à 18H00)

SYNOPSIS

**PROMOTEUR**	**FFCD**
**COORDONNATEUR**	Dr DAHAN Laetitia<br>Unité d'Oncologie Digestive<br>CHU TIMONE Adulte<br>264 rue St Pierre<br>13385 Marseille Cedex 05
**TITRE**	**PHARMACOGENETIQUE DE LA GEMCITABINE : ETUDE DE L'IMPACT DU POLYMORPHISME GENETIQUE DE LA CYTIDINE DEAMINASE (CDA) SUR LA TOXICITE DANS LES ADENOCARCINOMES PANCREATIQUES RESEQUES**
**PATHOLOGIE**	Adénocarcinome du pancréas opéré
**OBJECTIFS**	Objectif principal :<br>Evaluer la capacité du CDA à prédire la survenue d'une toxicité hématologique sévère (grade 3-4), précoce (lors des 2 premiers cycles), induite par la gemcitabine.<br>Objectifs secondaires :<br>- évaluer la capacité du CDA à prédire la survenue d'une toxicité non hématologique sévère (grade 3-4), précoce (lors des 2 premiers cycles), et lors des cycles suivants, induite par la gemcitabine ;<br>- évaluer la capacité du CDA à prédire la survenue d'une toxicité hématologique sévère (grade 3-4) durant l'ensemble des cycles, induite par la gemcitabine ;<br>- évaluer l'impact du statut CDA sur la pharmacocinétique de la gemcitabine et le ratio de métabolisation gemcitabine/dFdU ;<br>- étude génotype à phénotype sur le gène CDA ;<br>- recherche de nouvelles mutations sur le gène de la CDA.<br>Analyse exploratoire :<br>Evaluer la relation entre le statut CDA et la survie globale.
**SCHEMA D'ETUDE**	Etude multicentrique ouverte, non randomisée
**METHODOLOGIE**	La détermination du statut CDA se fera sur une base ***phénotypique*** (détermination spectrophotométrique de l'activité résiduelle sérique) et ***génotypique*** après extraction de l'ADN, selon des méthodes déjà disponibles et publiées (ex : HRM). La détermination phénotypique de la

	CDA a fait l'objet d'un dépôt de brevet.<br>La détermination des paramètres pharmacocinétiques (étude optionnelle) se fera après extraction liquide-liquide et dosage chromatographique par HPLC-UV de la gemcitabine et de son principal métabolite (dFdU), selon une méthode déjà publiée.
**CRITERES D'INCLUSION**	- Adénocarcinome du pancréas, histologiquement prouvé, avec résection chirurgicale à visée curative, macroscopiquement complète (R0 ou R1) et pour lequel un traitement adjuvant par gemcitabine seule pendant 6 mois est nécessaire (décision devant être validée dans une RCP)<br>- Age ≥ 18 ans<br>- Etat général OMS 0, 1 ou 2<br>- Pas de contre-indication à la gemcitabine<br>- Critères biologiques : PNN ≥ 1500/mm^3, plaquettes ≥ 100 000/mm^3, phosphatases alcalines ≤ 5N, bilirubine totale ≤ 50 µmol/L, clairance de la créatinine ≥ 60 mL/min<br>- Apte à débuter une chimiothérapie adjuvante dans les 8 semaines post-opératoires<br>- Consentement éclairé signé
**CRITERES DE NON INCLUSION**	- Tumeur du pancréas métastatique, ou localement avancée non résécable<br>- Ampullome, carcinome endocrine<br>- Syndrome infectieux évolutif (fièvre > 38°C ou abcès)<br>- Chirurgie d'exérèse ayant laissé des résidus tumoraux macroscopiques (R2)<br>- Chimiothérapie ou radiothérapie antérieures (dans les 10 ans)<br>- Antécédents de tumeur maligne autre qu'un carcinome basocellulaire cutané ou un épithélioma *in situ* du col utérin. Antécédents de tumeur maligne diagnostiquée et traitée de plus de 10 ans autorisés, sauf cancer du sein et mélanome<br>- Femme enceinte, susceptible de l'être ou en cours d'allaitement
**DEROULEMENT DE L'ETUDE**	▪ **Deux prélèvements sanguins** (1 tube EDTA de 10 mL + 1 tube sec de 5 mL) sont réalisés **avant la 1ère administration** de la chimiothérapie. Les 2 tubes seront gardés à 4°C jusqu'à la centrifugation ou le transfert en cryotube. La fraction sérique (tube sec) et le sang total (tube EDTA) seront conservés à -80°C jusqu'à

	acheminement en deux envois groupés. Ces prélèvements permettront d'une part la détermination du phénotype CDA (dosage de l'activité résiduelle sérique par spectrométrie visible, tube sec) et d'autre part feront l'objet d'analyses génétiques après extraction de l'ADN (tube EDTA : techniques HRM).<br>▪ Administration d'une cure standard de gemcitabine selon les référentiels en vigueur.<br>▪ Prélèvements plasmatiques pour l'établissement des **profils pharmacocinétiques (étude optionnelle)** durant la première administration de gemcitabine (n=4 tubes, T0, Tfin, Tfin+90 min, Tfin+120 min). Ces tubes seront centrifugés et la fraction plasmatique conservée à -80°C jusqu'à acheminement en deux envois groupés. Après extraction liquide/liquide, le dosage de la gemcitabine plasmatique et de son principal métabolite (dFdU) sera réalisé par chromatographie (HPLC-UV). L'établissement des paramètres pharmacocinétiques individuels se fera selon les techniques de modélisation compartimentale usuelle. Le ratio de métabolisation gemcitabine/dFdU sera calculé.<br>▪ Suivi des toxicités (NCI CTC V4.0) durant toute la durée du traitement.<br>▪ Surveillance hebdomadaire de la NFS et toutes les 12 semaines un bilan biologique complet et une imagerie (TDM abdominale ou IRM abdominale ou échographie abdominale) selon les habitudes du centre investigateur (tous les 3 ou 6 mois).
**EFFECTIF DE L'ESSAI**	Sur la base des données déjà recueillies (prévalence de 12,5% des toxicités hématologiques précoces grade 3 et 4), pour mettre en évidence un ratio de vraisemblance positif du statut CDA supérieur à 3,5 − objectif atteint, entre autres, lorsque la sensibilité est supérieure à 70 % et la spécificité est supérieure à 80 % − il est requis d'inclure 107 patients. 10 % de patients supplémentaires seront inclus pour tenir compte des perdus de vue et des patients n'ayant pas reçu au moins deux cycles de chimiothérapie, soit un total de 120 patients.
**ECHÉANCIER**	Durée des inclusions estimée: 24 mois.<br>Durée de l'étude prévue: 3 ans.<br>Nombre de patients estimé: 120.

1. INTRODUCTION ET JUSTIFICATION DE L'ETUDE

Le cancer du pancréas touche environ 5 000 nouveaux patients chaque année en France et représente la quatrième cause de mortalité par cancer. Le traitement curatif reste la chirurgie, mais seuls 10% des patients sont effectivement opérables. Le traitement de ce cancer est donc le plus souvent palliatif et le pronostic demeure très sombre (survie médiane, tous stades confondus : 6 mois). La chimiothérapie de référence, que ce soit pour le traitement adjuvant après résection à visée curative, ou en situation palliative en première ligne, reste la gemcitabine en monothérapie. Bien que ce traitement soit généralement bien toléré par une majorité de patients, 5 à 10% d'entre eux présentent des toxicités sévères (principalement hématologiques et infectieuses) au cours du premier ou du second cycle de traitement. La survenue de ces toxicités ne semble pas liée à un facteur connu (âge, sexe, fonction rénale ou hépatique) et demeure ainsi totalement imprévisible. Par ailleurs, l'efficacité de la chimiothérapie dans les adénocarcinomes pancréatiques demeure relative, avec une médiane de survie n'excédant pas 6 mois chez les patients métastatiques. En situation adjuvante chez les patients opérés, l'administration de gemcitabine pendant 6 mois double la médiane de survie sans rechute (13,4 vs 6.9 mois – p<0.001) et le taux de survie à 5 ans (22,5 vs 11,5 %) par rapport à la chirurgie seule (Oettle, JAMA 2007).

La détoxification de la gemcitabine est sous la dépendance d'une enzyme unique, la cytidine déaminase (CDA) hépatique, faisant l'objet d'un polymorphisme génétique responsable d'une grande variabilité de fonctionnalité enzymatique (déficience ou hypermétabolisation). Il a été récemment suggéré que l'administration d'une dose standard de gemcitabine chez un patient déficient en CDA pouvait induire une surexposition plasmatique en gemcitabine, potentiellement responsable des toxicités sévères, voire létales rapportées. Inversement, les faibles réponses observées avec la gemcitabine pourraient être en partie dues à une surcapacité métabolique (phénotype extensif) chez certains patients. Disposer d'un outil de typage permettant d'évaluer le statut « CDA » des patients devrait ainsi permettre une meilleure individualisation thérapeutique de la gemcitabine et une optimisation de la prise en charge par chimiothérapie des patients concernés, en intégrant comme variable d'ajustement leur capacité métabolique à détoxifier correctement ce médicament. La mise en place de stratégies de typage pour améliorer et individualiser la prise en charge par chimiothérapie est actuellement en plein essor (Maitland et al. Pharmacogenetics 2006). Ce projet s'inscrit dans cette tendance à développer une médecine personnalisée en oncologie clinique.

2. DONNEES DE LA LITTERATURE ET PRE-REQUIS

Le polymorphisme génétique affectant le gène codant pour la CDA a fait l'objet de diverses études fondamentales et les premières mutations (SNPs) ont été décrites depuis une quinzaine d'années (Kirch et al. Exp Hematol 1998, Gilbert et al., Clin Cancer Res 2006). Aujourd'hui encore, l'ensemble des mutations affectant ce gène demeurent loin d'être connues, ainsi que le rôle d'éventuelles dysrégulations épigénétiques. Le projet actuel repose sur l'hypothèse que le statut CDA impacte sur les niveaux d'exposition plasmatique à la gemcitabine et sur le risque de survenue de surtoxicités chimio-induites (patients déficients) ou d'échappement thérapeutique (patients métaboliseurs extensifs). Diverses équipes se sont très récemment attachées à déterminer la relation entre l'activité CDA et les toxicités induites par la gemcitabine (Yonemori et al. clin Cancer Res 2005, Tibaldi et al., Clin Cancer Res 2008, Matsubara et al., J Clin Oncol 2009), essentiellement sur la base d'une expertise génotypique. La mutation 208G>A (CDA*3) a ainsi été fréquemment évoquée, au sein de la population japonaise, comme potentiellement reliée à une perte fonctionnelle avec survenue d'une toxicité hématologique sous gemcitabine (Yonemori et al. J Clin Oncol 2007, Ueno et al. Br J Cancer 2009). Toutefois, ce marqueur de prédisposition semble être uniquement retrouvé au sein de la seule population asiatique, voire uniquement au Japon, (Mercier et al. J Clin Oncol 2007). De façon similaire, la mutation 79A>C a été récemment désignée comme possible facteur prédictif de toxicité et de survie chez des patients caucasiens atteints de carcinome des VADS traités par une combinaison gemcitabine + sel de platine (Tibaldi et al. Clin Cancer Res 2008, Giovanetti et al., Nucleosides Nucleotides Nucleic Acids 2009). Les relations génotype-à-phénotype avec la mutation 79A>C sont en fait loin d'être univoques, et des données contradictoires sur son impact fonctionnel rendent le recours à sa recherche comme marqueur prédictif délicat (Yue et al. Pharmacogenetics 2003). En raison du manque de consensus sur l'impact réel des mutations génétiques sur le phénotype CDA, une approche fonctionnelle apparaît comme une stratégie plus judicieuse pour déterminer le statut CDA d'un patient donné. Notre groupe a développé un outil de détection permettant de déterminer l'activité résiduelle sérique en CDA comme marqueur indirect de l'activité de détoxification. Cet outil a d'abord été validé sur un modèle animal qui a démontré que ce test permettait effectivement d'identifier des animaux déficients en CDA (André et al. Proceedings of the AACR 2008). Chez ces animaux, la déficience en CDA s'est traduite par des toxicités hématologiques létales à type de neutropénie profonde, du fait d'une surexposition plasmatique après administration d'une dose standard de gemcitabine, apportant ainsi une première preuve de concept. Nous avons en effet démontré dans cette étude que le déficit en CDA se traduisait chez l'animal par une altération des paramètres pharmacocinétiques de la gemcitabine (diminution de la clairance plasmatique de $1,26.10^{-2}$ à $3,25.10^{-3}$ L/min/kg, diminution de l'index de métabolisation gemcitabine-dFdU d'un facteur 18). La

surexposition plasmatique résultante (142 vs 54 µg/mL/min) s'est systématiquement traduite par un décès toxique. De façon notable, l'utilisation de notre test fonctionnel, avant de débuter la chimiothérapie, a pleinement permis d'identifier les souris déficientes en CDA des souris normales. Dans une deuxième étape, ce test a été appliqué dans le cadre d'une étude clinique de faisabilité chez 130 adultes et 20 enfants traités par de la gemcitabine pour diverses affections cancéreuses. Au sein de cette population, 12% des patients ont connu un épisode toxique précoce sévère (> grade 3, CTC) après administration de la gemcitabine. Le statut CDA (phénotype et recherche des mutations ''simple permutation'' usuellement décrites : 79A>C, 208G>A, 435T>C) a ainsi été évalué rétrospectivement chez ces patients. L'activité CDA moyenne était de 3,5 U/mg et suivait une distribution normale. Le seul critère associé à la survenue des toxicités sévères précoces a été une activité CDA < 1,2 U/mg chez les patients traités par monothérapie et 1,4 U/mg chez les patients sous polychimiothérapie, suggérant l'existence d'un seuil associé à un risque accru de toxicité chimio-induite (Dahan et al., Proceedings of the Asco-GI, 2009 et Ciccolini et al., J Clin Oncol 2010). Cette étude a en outre mis en évidence l'absence de relations génotype-à-phénotype claires, confirmant l'intérêt de l'approche phénotypique. En effet, le statut homozygote ou hétérozygote pour les mutations 79A>C, 435T>C et 208 G>A n'a pas pu être relié à la survenue des toxicités observées. Notre groupe a en outre publié le premier cas mondial de décès toxique sous gemcitabine lié à un déficit en CDA, relié cette fois a une hétérozygotie en 79A>C, soulignant ainsi la difficulté d'isoler un statut génotypique prédictif (Ciccolini et al., Proceedings of the AACR 2007 et Mercier et al., Pharmacogenetics 2007). Nous avons également observé au cours de nos études préliminaires que les patients présentant un phénotype extensif (activité CDA> 6 U/mg, 10% des patients) présentaient des taux de réponse très inférieurs au reste de la population, suggérant un risque accru d'échappement thérapeutique par sur-métabolisation du médicament, mais compte tenu de l'hétérogénéité des traitements et des affections regroupées dans cette étude préliminaire, il ne nous a pas été encore possible d'établir une corrélation entre ces deux évènements (Dahan et al., Proceedings of the Asco-GI, 2010).

3. OBJECTIFS DE L'ETUDE

L'objectif de cette étude est de démontrer la pertinence d'une stratégie de typage (phénotype/génotype) de la CDA comme marqueur prédictif de surexposition ou sous-exposition plasmatique en gemcitabine, avec survenue de toxicités sévères précoces. Une expertise pharmacocinétique doit objectiver les variations d'exposition plasmatique au médicament et un déficit de métabolisation (suivi du ratio de conversion gemcitabine-dFdU). La tolérance sera monitorée au travers des toxicités précoces et sur l'ensemble des cycles. Cette étude permettra de valider ou d'affiner les seuils identifiés durant l'étude de faisabilité sur une population hétérogène.

Au final, cette étude devrait permettre de démontrer si la CDA peut-être considérée comme une variable d'ajustement de la posologie de la gemcitabine. Secondairement, au cours d'une autre étude, il sera alors possible de proposer prospectivement un typage CDA chez tout patient devant recevoir de la gemcitabine afin d'établir sa capacité à détoxifier cette molécule, évaluer le risque toxique chimio-induit ou de non-réponse, et, en cas de risque avéré, proposer une adaptation posologique en accord avec le statut métaboliseur du patient. Cette stratégie devrait permettre de proposer un outil autorisant une meilleure individualisation et sécurisation du traitement par la gemcitabine, troisième anticancéreux le plus prescrit au monde.

3.1 Objectif Principal

Evaluer la capacité du CDA à prédire la survenue d'une toxicité hématologique sévère (grade 3-4), précoce (lors des 2 premiers cycles), induite par la gemcitabine.

3.2 Objectifs Secondaires

- évaluer la capacité du CDA à prédire la survenue d'une toxicité non hématologique sévère (grade 3-4), précoce (lors des 2 premiers cycles) et sur l'ensemble des cycles, induite par la gemcitabine ;
- évaluer la capacité du CDA à prédire la survenue d'une toxicité hématologique sévère (grade 3-4) durant l'ensemble des cycles, induite par la gemcitabine ;
- évaluer l'impact du statut CDA sur la pharmacocinétique de la gemcitabine et le ratio de métabolisation gemcitabine/dFdU ;
- étude génotype à phénotype sur le gène CDA ;
- recherche de nouvelles mutations sur le gène de la CDA.

3.3 Analyse exploratoire

Il sera étudié, au cours d'une analyse exploratoire, la relation entre le statut CDA et la survie globale.

4. CRITERES DE SELECTION

Il s'agit d'une étude prospective, multicentrique ouverte, non randomisée.

120 patients adultes, n'ayant pas reçu de chimiothérapie antérieure, devant recevoir de la gemcitabine en monothérapie, en traitement adjuvant d'un adénocarcinome pancréatique réséqué, sont prévus à l'inclusion.

4.1 Critères d'inclusion

- Adénocarcinome du pancréas, histologiquement prouvé ; patient ayant eu une résection chirurgicale à visée curative, macroscopiquement complète (R0 ou R1) et

pour lequel un traitement adjuvant par gemcitabine seule pendant 6 mois est nécessaire (décision devant être validée dans une RCP)

- Age ≥ 18 ans
- Etat général OMS 0, 1 ou 2
- Pas de contre-indication à la gemcitabine
- Critères biologiques : PNN ≥ 1500/mm^3, plaquettes ≥ 100 000/mm^3, phosphatases alcalines ≤ 5N, bilirubine totale ≤ 50 µmol/L, clairance de la créatinine ≥ 60 mL/min
- Apte à débuter une chimiothérapie adjuvante dans les 8 semaines post-opératoires
- Consentement éclairé signé

4.2 Critères de non inclusion

- Tumeur du pancréas métastatique ou localement avancée non résécable
- Ampullome ou carcinome endocrine
- Syndrome infectieux évolutif (fièvre > 38 °C ou abcès)
- Chirurgie d'exérèse ayant laissé des résidus tumoraux macroscopiques (R2)
- Chimiothérapie ou radiothérapie antérieures (dans les 10 ans précédant l'inclusion)
- Antécédents de tumeur maligne autre qu'un carcinome basocellulaire cutané ou un épithélioma *in situ* du col utérin ; antécédents de tumeur maligne diagnostiquée et traitée de plus de 10 ans autorisés, sauf cancer du sein ou mélanome
- Femme enceinte, susceptible de l'être ou en cours d'allaitement

5. BILAN INITIAL

La visite de pré-inclusion, dans les 7 jours précédant l'inclusion, doit comporter:

- un examen clinique complet avec mesure du poids, de la taille, de la surface corporelle, estimation de l'état général OMS, anamnèse et médicaments associés ;
- un bilan biologique comprenant :
  - NFS-plaquettes ;
  - CRP ;
  - Ionogramme sanguin (sodium, potassium), glycémie, créatininémie avec mesure de la clearance de la créatinine selon la formule de Cockroft [chez l'homme: (140-âge) x poids en kg/0.814 x créatinine en µmol et chez la femme: (140-âge) x poids en kg/0.85 x créatinine en µmol] et urée ;
  - Bilan hépatique comprenant ASAT, ALAT, PAL, bilirubine totale et conjuguée, TP, albuminémie ;
  - CA19-9 sérique ;

- ECG systématique, avis cardiologique en cas d'antécédents de cardiopathie ischémique ;
- Radiographie thoracique ;
- Scanner abdominal (si l'examen préopératoire date de plus de 12 semaines) ;

6. INCLUSION

Après signature du consentement et validation des critères de sélection, une fiche d'inclusion disponible dans le cahier d'observation sera faxée par l'investigateur **au 03 80 38 18 41**.

Le Centre de Randomisation - Gestion -Analyse de la FFCD à Dijon est ouvert du lundi au vendredi de 8h00 à 18h00. Le numéro d'enregistrement sera transmis au médecin investigateur.

7. PRELEVEMENTS SANGUINS POUR LES ETUDES PHARMACOGENETIQUES ET PHARMACOCINETIQUES

7.1 Etude pharmacogénétique et phénotypique du CDA (obligatoire) :

Deux prélèvements sanguins (1 tube EDTA + 1 tube sec) devront être réalisés avant la première administration de la gemcitabine. Ces tubes permettront d'une part la détermination du phénotype CDA (tube sec avec ou sans activateur de coagulation : dosage de l'activité résiduelle sérique par spectrométrie visible) et d'autre part feront l'objet d'analyses génétiques après extraction de l'ADN (tube EDTA : technique PCR HRM (High Resolution Melting)).

Phénotype CDA : Le tube sec (bouchon rouge – 5 mL) sera stocké à 4°C (réfrigérateur) immédiatement après prélèvement et rapidement centrifugé (4°C, 2500 rpm, 20 mn) avec un délai maximum de 10 heures entre prélèvement et centrifugation. Après isolement de la fraction sérique dans un cryotube, l'échantillon sera stocké à -80°C dans le centre et le culot cellulaire sera détruit.

Génotype CDA : Le tube EDTA (bouchon mauve – 10 mL) sera stocké à 4°C immédiatement après prélèvement avant d'être transféré dans un cryotube qui sera stocké à -80°C dans le centre jusqu'à acheminement.

La fraction sérique et le sang total pourront être acheminés conjointement. Il y aura 2 envois groupés des prélèvements, le 1er à mi-parcours et le 2ème en fin d'étude. Les prélèvements groupés seront acheminés par transporteur (centres hors-APHM) ou par coursier interne (centres APHM) sous carboglace au Laboratoire de Transfert en Oncologie du Pôle Oncologie de l'APHM (Médecine Nord, Pr L.H. Ouafik Bd Pierre Dramart, 13015 Marseille Tel : 04 91 69 88 82/81).

Dès réception, la fraction sérique sera stockée à -80°C jusqu'à l'analyse phénotypique ; elle fera l'objet d'une évaluation fonctionnelle de l'activité CDA par spectrophotométrie visible et d'un dosage des protéines sériques par la méthode de Bradford.

Le sang total fera l'objet d'une extraction de l'ADN lymphocytaire selon les procédures usuelles. Les ADN seront ensuite stockés à 4°C jusqu'à analyse. Les échantillons d'ADN feront l'objet d'un typage par HRM (High Resolution Melting PCR, technologie Light Cycler) afin de rechercher le polymorphisme 79A>C et toute autre anomalie génétique.

Tube sec (rouge) → stocké au frigo puis centrifugé dans le centre (dans les 10h) → Isolement de la fraction sérique dans un cryotube ↓

Stockage à -80°C dans le centre → 2 envois groupés en carboglace par DHL au labo de transfert (Marseille)

Tube EDTA (mauve) → stocké au frigo dans le centre → Transfert du sang total dans un cryotube ↓

Stockage à -80°C dans le centre → 2 envois groupés en carboglace par DHL au labo de transfert (Marseille)

Constitution d'une collection liée à l'étude de la CDA (CDA-laboratoire de Transfert, Marseille) : Les échantillons seront conservés à -80°C (sérums) et 4°C (ADN) jusqu'à la conclusion de l'étude. Les sérums seront détruits après analyse. Les échantillons d'ADN seront conservés à des fins exploratoires éventuelles, en fonction de l'évolution des connaissances scientifiques. Ils pourront faire l'objet d'une destruction ultérieure après avis du Comité de la Recherche Biologique de l'étude.

7.2 Etude pharmacocinétique (optionnelle) :

Ces recueils sont optionnels et se feront dans les centres identifiés et volontaires. Les 4 prélèvements plasmatiques pour l'établissement des profils pharmacocinétiques seront réalisés lors de la première perfusion de chimiothérapie (n=4 tubes, T0, Tfin, Tfin+90 min, Tfin+120 min). Les recueils PK se feront sur des tubes spécifiques coatés à la tetra-hydro-uridine (THU). Ces tubes seront préalablement fournis par le CHU Timone à l'ouverture des centres souhaitant participer à l'étude pharmacocinétique. Les tubes seront centrifugés (4°C, 2500 rpm, 20 mn), la fraction

plasmatique sera isolée et stockée à -80°C jusqu'à acheminement. Les échantillons seront acheminés sous carboglace par transporteur (centres hors-APHM) ou par coursier interne (APHM) au Laboratoire de Pharmacocinétique (Pr B. Lacarelle, CHU Timone, 264 rue St Pierre 13385 Marseille 05 Tel 04 91 38 75 68). L'acheminement pourra se faire conjointement avec les tubes de l'étude principale. Dès réception au laboratoire de Pharmacocinétique, les échantillons seront isolés et stockés à -80°C. Après extraction liquide/liquide, le dosage de la gemcitabine plasmatique et de son principal métabolite (dFdU) sera réalisé par chromatographie HPLC-UV phase inverse en utilisant la 5-flurocytosine comme étalon interne.

Devenir de cette collection d'échantillons après analyse (PK -laboratoire de pharmacocinétique, Marseille) : Les échantillons (plasma) seront conservés à -80°C durant toute la durée de l'étude et éliminés à la conclusion de celle-ci.

8. TRAITEMENT PAR GEMCITABINE – ADAPTATION DES DOSES

La prise en charge thérapeutique et le suivi des patients se font selon les recommandations habituelles. L'exérèse chirurgicale et l'analyse histologique de la pièce d'exérèse seront réalisées dans chaque centre selon les référentiels en vigueur ; les marges de résection de la tumeur seront analysées (R0, R1). La chimiothérapie adjuvante par la gemcitabine, validée par une RCP, doit débuter chaque fois que possible dans les 8 semaines post-opératoires ; elle comporte 6 cycles de 4 semaines (perfusions intraveineuses hebdomadaires de 1000 mg/m^2 en 30 minutes, 3 semaines sur 4) selon les recommandations.

8.1 Traitement

La gemcitabine sera disponible sous la forme d'une poudre lyophilisée dans des ampoules stériles de 200 mg ou de 1000 mg.

L'administration de la gemcitabine se fera selon les référentiels en vigueur, après décision de RCP :

- Prémédication par un Anti-5HT3 (1 ampoule intraveineuse) +/- associée à du Solumedrol ®
- **Gemzar® (gemcitabine) : 1000 mg/m²** à diluer dans 125 mL de sérum physiologique et à perfuser en intraveineux en 30 minutes
- Six cycles de 4 semaines sont prévus, avec cures hebdomadaires 3 semaines sur 4 (à J 1, J 8 et J 15), la quatrième semaine étant de repos
- Le Gemzar® étant utilisé dans le cadre de son AMM, il sera fourni par chaque centre investigateur. Le protocole d'administration du médicament et sa fiche Vidal correspondante figurent en annexe 1.

8.2 Adaptations de doses

L'adaptation de doses du Gemzar® se fera en fonction de la toxicité maximale.
Les toxicités seront évaluées avant chaque perfusion de gemcitabine, par l'interrogatoire et la biologie; elles seront gradées selon les critères NCI-CTC version 4.0 (annexe 3).
En cas de toxicité persistante le jour théorique de la cure, il faut attendre la récupération, avant de reprendre le traitement à dose adaptée.

- Toxicités hématologiques

Type de toxicité		Grade 0	Grade 1	Grade 2	Grade 3	Grade 4
**PNN** (/mm^3)	Nb	>2000	1 500-2 000	1 000-1 500	500-1 000	< 500
	Administration à la cure suivante	**100%**		0 (attente de récup>1500), puis **100 %** de la dose initiale	0 (attente de récup>1500), puis **75 %** de la dose initiale	0 (attente de récup>1500), puis **75 %** de la dose initiale
**Plaquettes** (/mm^3)	Nb	>100 000	75 000-100 000	50 000 - 75 000	25 000 - 50 000	< 25 000
	Administration à la cure suivante	**100 %**		0 (attente de récup>75000), puis **75 %** de la dose initiale	0 (attente de récup>75000), puis **75 %** de la dose initiale	0 (attente de récup>75000), puis **75 %** de la dose initiale

- Toxicités non hématologiques

Type de toxicité		Grade 0	Grade 1	Grade 2	Grade 3	Grade 4
Nausée/ vomissements	Administration à la cure suivante	100 %				Arrêt définitif
Autres	Administration à la cure suivante	100 %			50 %	Arrêt définitif

9. EVALUATION DE LA SECURITE

L'évaluation de la sécurité se fera par l'évaluation de l'état général, clinique et biologique des patients au moment des consultations prévues et par le recueil des événements survenant entre ces consultations. Les toxicités seront évaluées grâce à l'échelle de toxicité NCI-CTCAE version 4.0 (cf. annexe 3).

9.1 Définitions

Evénement Indésirable (EI)

Un événement indésirable est une manifestation nocive survenant chez une personne qui se prête à une recherche biomédicale, que cette manifestation soit liée ou non à la recherche ou au produit sur lequel porte cette recherche

Tous les événements indésirables seront consignés dans le cahier d'observation aux pages prévues à cet effet.

Evénement Indésirable Grave (EIG)

Est considéré comme un événement indésirable grave (EIG) tout évènement :

- Entraînant le décès,
- Mettant en jeu le pronostic vital,
- Entraînant une hospitalisation ou une prolongation d'hospitalisation,
- Provoquant une invalidité permanente ou une incapacité temporaire grave,
- Provoquant une anomalie congénitale, une malformation fœtale ou un avortement,
- Médicalement significatif.

Effet Indésirable

Toute réaction nocive et non désirée à un médicament expérimental quelle que soit la dose administrée ou à une préparation de thérapie cellulaire ou à tout élément expérimental. L'effet indésirable est grave s'il présente un critère de gravité (cf ci-dessus).

Effet Indésirable Grave Inattendu

Un effet indésirable grave inattendu est un événement non mentionné, ou différent par sa nature, son intensité, son évolution par rapport au document de référence du produit.

Dans cet essai, le document de référence est le Résumé des Caractéristiques du Produit (RCP) du GEMZAR® (cf. annexe 1).

La version du RCP utilisé sera la dernière disponible à la date anniversaire du début de l'essai.

9.2 Conduite à tenir en cas d'événement indésirable grave

L'investigateur informe le promoteur de tous les Evènements Indésirables Graves (attendus et inattendus) qu'ils soient imputables ou non à la recherche, qui se produisent pendant le traitement et dans les 30 jours suivant la dernière administration du traitement.

Tous les Evénements Indésirables Graves retardés (survenant après cette période de 30 jours) considérés comme raisonnablement liés au traitement protocolaire ou à la recherche doivent être déclarés sans limitation de délai.

La déclaration d'un EIG se fait dans les 24 heures ouvrées suivant sa constatation, en faxant au CRGA de la FFCD au 03 80 38 18 41, la fiche d'EIG datée, signée et documentée le plus précisément possible.
Des compléments d'informations pourront être demandés (par fax, par téléphone ou lors d'une visite) par l'attaché de recherche clinique et/ ou le promoteur.

Modalités et durée du suivi des patients, suite à la survenue d'événements indésirables :

L'investigateur est responsable du suivi médical approprié des patients, jusqu'à la résolution ou la stabilisation de l'effet ou jusqu'au décès du patient. Cela peut impliquer parfois que ce suivi se prolonge après la sortie d'étude du patient.

Il transmet les informations complémentaires au promoteur à l'aide d'un formulaire de déclaration des EIG (en cochant la case suivi n° X pour préciser qu'il s'agit d'un rapport de suivi et non d'un rapport initial) dans les 24 heures suivant leur obtention. Il transmet également le dernier suivi à la résolution ou à la stabilisation de l'EIG.

Il conserve les documents concernant l'effet indésirable présumé afin de permettre, en cas de nécessité, de compléter les informations précédemment transmises.

10. SUIVI DES PATIENTS

- **Surveillance pendant la chimiothérapie par la gemcitabine**
  Toutes les semaines sauf la semaine de repos :
  - poids, taille, état général OMS, données de toxicité clinique (NCI-CTC version 4.0), traitements associés ;
  - NFS, plaquettes.

- **A la demande en cas de symptômes**

- **Toutes les 12 semaines après inclusion, pendant et après l'arrêt du traitement adjuvant :**
  - examen clinique : poids, taille, état général OMS, traitements associés ;
  - toxicités NCI-CTC version 4.0 (jusqu'à la visite de fin de traitement, 30 jours après la dernière cure);

- biologie : NFS, plaquettes, ionogramme sanguin (Na+, K+), glycémie, créatininémie, clairance de la créatinine, urée, bilirubine totale et conjuguée, phosphatases alcalines, ALAT, ASAT, TP, albuminémie, CA 19-9;
- Imagerie (TDM abdominale ou IRM abdominale ou échographie abdominale), selon les habitudes du centre investigateur (tous les 3 ou 6 mois).

Tous les examens, prélèvements et dosages (sauf les analyses pharmacogénétiques et pharmacocinétiques) seront réalisés dans chaque centre investigateur. Il s'agit d'examens réalisés en routine hors essai dans les centres pratiquant la cancérologie digestive.

11. CRITERES DE JUGEMENT

Tolérance

Les critères suivants seront évalués selon les critères du NCI-CTC version 4.0 (cf annexe 3):

- toxicités hématologiques sévères (grade 3-4), évaluées à la fin de chacun des deux premiers cycles de traitement (objectif principal) ;
- ensemble des toxicités, sur toutes les cures (objectifs secondaires) ;
- temps jusqu'à apparition d'une toxicité hématologique de grade 3-4, défini comme l'intervalle de temps entre la date d'inclusion et la date de première apparition d'une toxicité hématologique de grade 3-4 ; les patients décédés ou perdus de vue n'ayant pas présenté de toxicité de grade 3-4 seront censurés à la date de décès ou à la date de point (ou à la date de dernières nouvelles si elle est antérieure) ; l'analyse sera répétée pour le temps jusqu'à apparition d'une toxicité de grade 3-4 ;
- incidence des événements indésirables graves ;
- évolution de l'indice de performance OMS et du poids ;
- description des doses, des adaptations de dose, et des retards de cure.

Statut CDA

Il s'évaluera suivant deux méthodes distinctes :

→ Un test fonctionnel de type dosage enzymatique par spectrophotométrie visible sur fraction sérique (Mercier et al., Pharmacogenetics 2007).

→ Une recherche de mutations sur le gène CDA : mutation 79A>C par technique de PCR (polymerase chain reaction) quantitative de type HRM (Ciccolini et al., J Clin Oncol 2010) et la recherche de toute autre mutation par HRM sur le gène de la CDA.

Dosage des taux plasmatiques de gemcitabine et de son métabolite

Il sera effectué par chromatographie HPLC-UV en phase inverse après extraction liquide-liquide selon la méthodologie décrite dans la littérature (André et al. AACR 2008).

Modèle pharmacocinétique/pharmacodynamique

Il sera bâti selon une approche stochastique comme décrite précédemment (Ciccolini et al. J Clin Oncol 2010) par le groupe de Pharmacométrie du Laboratoire de Pharmacocinétique de l'Université d'Aix-Marseille (Pr Athanassios Iliadis, UFR Pharmacie, 27 Bd Jean Moulin 13385 Marseille 05).

Survie globale (analyse exploratoire)

Analyse de l'intervalle de temps entre la date d'inclusion et la date de décès (quelle qu'en soit la cause). Les patients vivants ou perdus de vue seront censurés à la date de point, ou à la date de dernières nouvelles si elle est antérieure.

12. NOMBRE DE PATIENTS NECESSAIRES

12.1 Données de la littérature

Les hypothèses pour le calcul du nombre de sujets nécessaires reposent sur les données de l'article de Ciccolini et al. (2010), dans lequel les toxicités hématologiques sévères (grade 3-4), précoces, ont été relevées chez 64 patients en monothérapie. Un seuil de CDA de 1,30 U/mg a été identifié.

La capacité du statut CDA à prédire la survenue d'une toxicité sévère sera évaluée en fonction de ce seuil :

- une valeur de CDA inférieure à 1,30 U/mg serait associée à la survenue de toxicités sévères ;
- une valeur de CDA supérieure à 1,30 U/mg rejetterait la survenue de toxicités sévères.

Les données de l'article de Ciccolini et al., servant au calcul du nombre de sujets nécessaires, sont récapitulées dans le tableau suivant :

		**CDA**		
		**< 1,3 U/mg**	**> 1,3 U/mg**	Total
**Toxicité grade 3 - 4**	**Non**	0	56	56 (87,5 %)
	**Oui**	7	1	8 (12,5 %)
		7 (11 %)	57 (89 %)	64

12.2 Calcul du nombre de sujets nécessaires

L'objectif de l'étude sera de montrer que le ratio de vraisemblance positif du CDA est supérieur à 3,5. Cet objectif est atteint, entre autres, lorsque la sensibilité est supérieure à 70 %, et la spécificité supérieure à 80 %.

Hypothèses :

- H0 : le ratio de vraisemblance positif n'est pas différent de 3,5 ;
- H1 : le ratio de vraisemblance positif est différent de 3,5 ; un ratio de 8 est attendu.

Un ratio de vraisemblance positif de 8 est atteint, entre autres, lorsque la sensibilité est d'au moins 80 %, et la spécificité d'au moins 90 %.

Avec un risque α bilatéral de 5 %, il est requis d'inclure un effectif de 107 patients pour mettre en évidence, avec une puissance de 80 %, un ratio de vraisemblance positif supérieur à 3,5.

L'analyse de l'objectif principal sera réalisée auprès des patients non perdus de vue ayant eu au moins les 2 premiers cycles de gemcitabine ; de fait, 10 % de patients supplémentaires seront inclus, soit un nombre total de patients de 120.

12.3 Compléments sur le ratio de vraisemblance positif

Le ratio de vraisemblance positif est le rapport de la sensibilité sur le complément de la spécificité. Il mesure par quel facteur l'odd (probabilité/(1-probabilité)) de toxicité est multiplié lorsque le CDA est inférieur à 1,3 :

(Odd sachant CDA < 1,3) = ratio de vraisemblance positif × (Odd sans information sur le CDA)

La probabilité de toxicité sans connaissance du CDA est estimée à 12,5 % ; si le ratio de vraisemblance positif est supérieur à 3,5, alors, lorsque le CDA est inférieur à 1,30 U/mg, la probabilité de toxicité prédite est supérieure à 33 %. Si le ratio de vraisemblance est de 8, la probabilité de toxicité prédite en cas de CDA inférieur à 1,30 U/mg est de 53 %.

13. GESTION DES DONNEES ET STATISTIQUES

13.1 Durée totale prévisionnelle de l'étude

La durée prévisionnelle totale est de 3 ans :

Années 1 à 3 :

- inclusions (24 mois) et suivi clinique minimum (toxicités, survie : 12 mois) ;
- détermination du statut CDA (phénotype, mutations génétiques) : 24 mois.
- Dosage pharmacocinétique : 24 mois.

13.2 Analyse statistique

Un plan d'analyse statistique détaillé sera rédigé dès le début de l'étude ; des modifications pourront être effectuées ultérieurement, mais plus après le gel de la base de données.

La gestion et l'analyse des données seront sous la responsabilité du responsable méthodologique du CRGA de la FFCD (Tél : 03 80 66 80 13 ; Fax : 03 80 38 18 41).

Un attaché de recherche clinique (ARC) pourra remplir les fiches du cahier d'observation sous contrôle de l'investigateur du centre. Un exemplaire des fiches du cahier d'observation sera adressé au CRGA de Dijon par l'investigateur. Le CRGA validera les données et enverra des demandes de correction à l'investigateur en cas d'erreurs ou de données incomplètes.

Un plan de data management sera rédigé avant le début des inclusions.

Critères généraux

L'analyse du critère principal, des critères secondaires de tolérance, et l'analyse exploratoire porteront sur l'ensemble des patients inclus ayant reçu au moins les deux premiers cycles de gemcitabine.

Les variables qualitatives seront décrites sous forme d'effectif et de pourcentage de la population concernée, avec leur intervalle de confiance à 95%. Les variables quantitatives seront décrites par la moyenne, l'écart-type, la médiane et l'écart interquartile selon leur type de distribution.

La capacité du CDA à prédire la survenue d'une toxicité sera mesurée au travers des ratios de vraisemblance positifs et négatifs, de la sensibilité, de la spécificité et des valeurs prédictives positives et négatives, avec leurs intervalles de confiance à 95 %.

Le temps jusqu'à apparition d'une toxicité hématologique de grade 3-4, ainsi que le temps jusqu'à apparition d'une toxicité de grade 3-4 seront estimés à partir de la méthode de Kaplan-Meier ; ils seront décrits par les courbes associées, et le temps médian ainsi que son intervalle de confiance à 95 %.

Validation ou recherche d'un nouveau seuil pour définir le statut CDA

La recherche du seuil optimal de CDA à retenir pour prédire l'apparition d'une toxicité sera basée sur la maximisation de la fonction d'utilité de ce test dans la population dans laquelle il est utilisé. Cette utilité tient compte des erreurs de classement, de la prévalence de la pathologie, et éventuellement des coûts et des bénéfices des erreurs de classement et des classements corrects en termes d'état de santé (Jund et al, 2005). S'il est possible de

modéliser la distribution du CDA, l'estimation du seuil optimal et de son intervalle de confiance sera effectuée en utilisant une méthode d'inférence Bayésienne (Subtil et al., 2010) ; dans le cas contraire, une estimation empirique du seuil optimal sera fournie par un algorithme de type Newton-Raphson.

Analyse exploratoire

La survie globale sera estimée selon la méthode de Kaplan-Meier dans les deux groupes de patients (CDA < 1,30 U/mg et CDA > 1,30 U/mg). Elle sera décrite par les courbes associées, les pourcentages de survie à différents temps et leur intervalle de confiance à 95 %. Le temps de suivi médian sera calculé par la méthode dite de Kaplan Meier inversée.

Les courbes de survie seront comparées par un test du Log-Rank. Les Hazard Ratio (HR) univariés seront estimés à l'aide de modèles de Cox univariés avec leur intervalle de confiance à 95%. Des modèles de Cox multivariés seront réalisés afin de rechercher un effet indépendant du statut CDA. Les variables pour lesquelles la valeur de p <0,10 lors de l'analyse univariée seront introduites dans le modèle de Cox multivarié. Les variables suivantes seront spécifiquement étudiées : statut CDA, âge, sexe, état général OMS, stade de la maladie, albuminémie, perte de poids, CRP. Les pré-requis d'utilisation du modèle de Cox (log linéarité et hypothèse de proportionnalité des taux) seront vérifiés.

Modélisation PK/PD – PK pop

L'établissement des paramètres pharmacocinétiques individuels se fera selon les techniques de modélisation compartimentale usuelle en utilisant une approche stochastique. Le ratio de métabolisation gemcitabine/dFdU sera calculé. Un modèle de population sera construit selon les recommandations en vigueur à l'aide des logiciels NonMem et Monolix. L'algorithme permettant la détermination de la DMT en fonction du statut CDA sera construit sur Matlhab. La modélisation PK/PD sera conduite par le groupe de Pharmacométrie du Pr Iliadis (Université d'Aix-Marseille).

14. COMITES

14.1 Comité Directeur

Un Comité Directeur sera mis en place. C'est le coordonnateur de l'étude, le Dr. Laëtitia Dahan, qui le présidera. Ce comité comprendra un représentant de chaque groupe coopératif participant, le président de la FFCD, le statisticien de la FFCD et le président du Comité de Recherche Biologique. Sa mission sera entre autres de prendre les décisions liées à la gestion de la recherche (amendement, clôture prématurée si besoin, …).

14.2 Comité de la Recherche Biologique

Un Comité de Recherche Biologique sera établi, sa mission étant de s'occuper des problèmes en rapport avec les prélèvements et la mise en banque ainsi que l'organisation de leur analyse. Le comité se réunira régulièrement et rendra compte de ses propositions au Comité Directeur. Chaque groupe participant au sein du Comité Directeur aura un membre votant; le poids des voix sera proportionnel aux effectifs recrutés par le groupe. Ce comité inclura entre autre le coordonnateur de l'étude et un biologiste ; il sera présidé par le Pr L'Houcine Ouafik.

15. ASPECTS LEGAUX ET ETHIQUES

15.1 Promoteur de l'étude

Cette étude a pour promoteur la Fédération Francophone de Cancérologie Digestive (FFCD). L'étude a été enregistrée sous le numéro EudraCT : 2010-022987-11.

15.2 Rappel des textes en vigueur

Cet essai se déroulera selon la Nouvelle Directive Européenne 2001/20/CE.

15.3 Assurance de responsabilité Civile

Une assurance a été souscrite par le promoteur auprès de HDI-GERLING sous le n° du contrat (1680) 90712 (Annexe 6), conformément à l'article L 1121-10 du code de la santé publique.

15.4 Demande d'autorisation au CPP et à l'AFSSAPS

Ce protocole a reçu l'avis favorable du CPP (Comité de Protection des Personnes) Sud Méditerranée I le 09/03/2011 (Annexe 7).
Ce protocole a reçu l'autorisation de l'AFSSAPS (Agence Française de Sécurité Sanitaire des Produits de Santé) le 31/01/2011(Annexe 8).

15.5 Recueil du consentement du malade

L'investigateur s'engage à recueillir, après information, le consentement clinique du patient par écrit (fiche d'information et formulaire de consentement en Annexe 2). Un double de ce consentement doit être conservé par l'investigateur pendant 15 ans, pour être présenté aux autorités de tutelle en cas d'inspection. L'original doit être remis au patient.

15.6 Information des directions hospitalières et convention de recherche

Préalablement à la mise en place de l'étude, les directions hospitalières seront informées par le promoteur de la participation à l'étude des investigateurs déclarés présents dans

leur(s) établissements(s), ainsi que le nom de l'investigateur principal concerné. Le promoteur adressera aussi une proposition de convention hospitalière sans surcoût ; entre l'administrateur du centre investigateur et le promoteur.

15.7 Archivage des données

Les dossiers resteront confidentiels et ne pourront être consultés que sous la responsabilité des médecins ayant en charge les patients. Le promoteur et les autorités sanitaires en cas d'inspection auront un accès direct à ces documents.
A la fin de l'essai, le cahier d'observation sera conservé pendant 15 ans par l'investigateur.

15.8 Support informatique

Conformément au texte de la loi n° 78-17 du 6 janvier 1978 modifiée par la loi du 9 août 2004, relative à l'informatique, aux fichiers et aux libertés, les données de l'essai seront enregistrées dans une banque de données informatiques du Centre de Randomisation et de Gestion Analyse de la FFCD, à l'exclusion des éléments relatifs à l'identité des malades. La FFCD a fait une déclaration simplifiée (MR01) auprès de la CNIL pour la mise en place de ses bases de données centralisées.

15.9 Traitement des données

Le Centre de Randomisation, de Gestion et d'Analyse (CRGA) de la FFCD sera responsable de la gestion et de l'analyse des données.

15.10 Monitorage, assurance qualité et inspections par les autorités

L'investigateur accepte par avance que les dossiers des patients inclus soient consultés par une personne mandatée par la FFCD et/ou par les autorités de santé pour procéder à un audit. Les visites de dossiers sur site, programmées après accord de l'investigateur pourront avoir lieu pendant ou après la période d'inclusion dans l'essai.
Ce protocole sera monitoré par les ARC mobiles de la FFCD suivant les bonnes pratiques cliniques. Une aide à la transcription pourra être apportée par les EMRC.

16. REGLES DE PUBLICATIONS

Elles seront conformes à celles établies par la FFCD (Annexe 5).

17. BIBLIOGRAPHIE

Andre N, Ortiz A, Mercier C, et al. Phenotypic determination of CDA status: Animal study and application in paediatric oncology. AACR Meeting Abstracts, Apr 2008; Los Angeles CA USA (abstract # 4806A).

Ciccolini J, Dahan L, André N, et al. Cytidine Deaminase Residual Activity in Serum Is a Predictive Marker of Early Severe Toxicities in Adults After Gemcitabine-Based Chemotherapies. J Clin Oncol. 28(1):160-5, 2010

Dahan L, Ciccolini J, Mercier C, Duluc M, Giacometti S, Evrard A, Seitz JF. A simple test to anticipate severe toxicities upon gemcitabine intake. 2009 ASCO Gastrointestinal Cancers Symposium, January 2009, Orlando FL USA (Abstract #151).

Gilbert JA, Salavaggione OE, Ji Y, et al. Gemcitabine pharmacogenomics: cytidine deaminase and deoxycytidylate deaminase gene resequencing and functional genomics. Clin Cancer Res. 12: 1794-803, 2006.

Giovannetti E, Laan AC, Vasile E, et al. Correlation between cytidine deaminase genotype and gemcitabine deamination in blood samples. Nucleosides Nucleotides Nucleic Acids. 27: 720-5, 2008.

Jund J, Rabilloud M, Wallon M, Ecochard R. Methods to estimate the optimal threshold for normally or log-normally distributed biological tests. Med Decis Making. 25(4):406-15, 2005.

Kirch HC, Schröder J, Hoppe H, et al. Recombinant gene products of two natural variants of the human cytidine deaminase gene confer different deamination rates of cytarabine in vitro. Exp Hematol. 26: 421-5, 1998.

Maitland ML, Vasisht K, Ratain MJ. TPMT, UGT1A1 and DPYD: genotyping to ensure safer cancer therapy? Trends Pharmacol Sci. 2: 432-7, 2006.

Mercier C, Raynal C, Dahan L, et al. Toxic death case in a patient undergoing gemcitabine-based chemotherapy in relation with cytidine deaminase downregulation. Pharmacogenet Genomics. 17: 841-4, 2007.

Mercier C, Evrard A, Ciccolini J. Genotype-based methods for anticipating gemcitabine-related severe toxicities may lead to false-negative results. J Clin Oncol. 25: 4855, 2007.

Subtil F, Rabilloud M. A Bayesian method to estimate the optimal threshold of a longitudinal biomarker. Biom J. 52(3):333-47, 2010.

Tibaldi C, Giovannetti E, Vasile E, et al. Correlation of CDA, ERCC1, and XPD polymorphisms with response and survival in gemcitabine/cisplatin-treated advanced non-small cell lung cancer patients. Clin Cancer Res. 14: 1797-803, 2008.

Ueno H, Kaniwa N, Okusaka Tet al. Homozygous CDA*3 is a major cause of life-threatening toxicities in gemcitabine-treated Japanese cancer patients. Br J Cancer. 100: 870-3, 2009.

Yen JL, McLeod HL. Should DPD analysis be required prior to prescribing fluoropyrimidines? Eur J Cancer. 43:1011-6, 2007.

Yue L, Saikawa Y, Ota K, et al. A functional single-nucleotide polymorphism in the human cytidine deaminase gene contributing to ara-C sensitivity. Pharmacogenetics. 13: 29-38, 2003.

ANNEXE 1 : fiche produit du GEMZAR® gemcitabine

FORMES et PRÉSENTATIONS

Lyophilisat pour usage parentéral IV à 200 mg :
Flacon (verre) de 10 ml.
Lyophilisat pour usage parentéral IV à 1 000 mg :
Flacon (verre) de 50 ml.

COMPOSITION

Lyophilisat à 200 mg :	p flacon
Gemcitabine (DCI)	200 mg
(sous forme de chlorhydrate : 228 mg/flacon)	

Lyophilisat à 1 000 mg :	p flacon
Gemcitabine (DCI)	1 000 mg
(sous forme de chlorhydrate : 1 140 mg/flacon)	

Excipients (communs) : mannitol, acétate de sodium, acide chlorhydrique, hydroxyde de sodium.

DC/INDICATIONS

La gemcitabine est indiquée dans le traitement de patients atteints de :

- Cancer bronchique non à petites cellules, localement avancé ou métastatique.
- Adénocarcinome du pancréas, localement avancé ou métastatique.
- Cancer de la vessie au stade invasif.

DC/POSOLOGIE et MODE D'ADMINISTRATION

Posologie :

Adulte :

- Cancer bronchique non à petites cellules :
  En monochimiothérapie :
  La dose recommandée est de 1 000 mg/m^2, administrée par une perfusion IV de 30 minutes. L'administration doit être répétée une fois par semaine pendant 3 semaines, suivie d'une semaine de repos. Ce cycle de 4 semaines sera alors renouvelé. Une réduction ou un report de la dose pourra être envisagé en fonction de la tolérance individuelle des patients.
  En association :
  La gemcitabine en association avec le cisplatine peut être administrée selon deux schémas posologiques; l'un est basé sur des cycles de 3 semaines, l'autre sur des cycles de 4 semaines.
  L'administration par cycles de 3 semaines est le schéma usuel ; le cycle de 3 semaines comprend une administration de 1 250 mg/m^2 de gemcibatine en perfusion IV de 30 minutes, les jours 1 et 8, suivie d'une semaine de repos pour un cycle de 21 jours. Ce cycle de 3 semaines sera alors renouvelé. Une réduction ou un report de la dose avant chaque administration de la chimiothérapie pourra être envisagé en fonction de la tolérance individuelle des patients.
  Le cycle de 4 semaines comprend une administration de 1 000 mg/m^2 de gemcitabine en perfusion IV de 30 minutes, les jours 1, 8 et 15, suivie d'une semaine de repos pour un cycle de 28 jours. Ce cycle de 4 semaines sera alors renouvelé. Une réduction ou un report de la dose avant chaque administration de la chimiothérapie pourra être envisagé en fonction de la tolérance individuelle des patients.
- Adénocarcinome du pancréas :
  La dose recommandée est de 1 000 mg/m^2, administrée en perfusion IV de 30 minutes. L'administration doit être répétée une fois par semaine pendant 7 semaines consécutives, suivie d'une semaine de repos. Puis, à partir du cycle suivant, l'administration doit être

répétée une fois par semaine pendant 3 semaines consécutives, suivie d'une semaine de repos. Les doses pourront être réduites avant chaque administration de la chimiothérapie, en fonction de la tolérance individuelle des patients à la gemcitabine.

- Cancer de la vessie au stade invasif :
  En association :
  La dose recommandée de gemcitabine, en association avec le cisplatine, est de 1 000 mg/m^2 en perfusion intraveineuse de 30 minutes les jours 1,8 et 15, suivie d'une semaine de repos pour un cycle de 28 jours. Le cisplatine est donné à la dose recommandée de 70 mg/m^2 à J2. Ce cycle de 4 semaines sera alors renouvelé. Une réduction ou un report de la dose avant chaque administration de la chimiothérapie pourra être envisagé en fonction de la tolérance individuelle des patients.
  Dans une étude clinique, en association au cisplatine à la dose de 100 mg/m^2, la myélosuppression a été plus importante.

- Quelle que soit l'indication :
  Les patients recevant de la gemcitabine doivent être soumis, avant chaque administration, à une surveillance hématologique : numération formule sanguine et plaquettes. Si nécessaire, la dose de gemcitabine sera adaptée comme indiqué ci-dessous :

Nombre absolu de granulocytes (x 10^6/l)	Nombre de plaquettes (x 10^6/l)	% de la dose totale
> 1 000 et > 100 000		100
500 - 1 000 ou 50 000 - 100 000		75
< 500 ou < 50 000		0

Un examen clinique et des contrôles périodiques des fonctions hépatique et rénale devront être faits afin de détecter une toxicité non-hématologique. Les doses pourront être réduites avant chaque administration de la chimiothérapie, en fonction de la tolérance individuelle des patients. Le traitement pourra être suspendu, suivant l'avis du médecin, jusqu'à résolution de la toxicité.
Patient âgé : la gemcitabine a été bien tolérée par les patients de plus de 65 ans. Les données pharmacocinétiques suggèrent que l'âge n'a pas d'effet sur le métabolisme du médicament.
Enfant : la gemcitabine n'a pas été étudiée chez les enfants.

Mode d'administration :

Voie intraveineuse stricte.
La gemcitabine est bien tolérée au cours de la perfusion et est généralement facile à administrer. Les cas de réaction au site de l'injection sont rares ; aucun cas de nécrose cutanée n'a été rapporté.
En cas d'extravasation, l'administration sera interrompue immédiatement.

La préparation des solutions injectables de cytotoxiques doit être obligatoirement réalisée par un personnel spécialisé et entraîné ayant une connaissance des médicaments utilisés, dans des conditions assurant la protection de l'environnement et surtout la protection du personnel qui manipule. Elle nécessite un local de préparation réservé à cet usage. Il est interdit de fumer, de manger, de boire dans ce local. Les manipulateurs doivent disposer d'un ensemble de matériel approprié à la manipulation, notamment blouses à manches longues, masques de protection, calot, lunettes de protection, gants à usage unique stériles, champs de protection du plan de travail, conteneurs et sacs de collecte des déchets. Les excreta et les vomissures doivent être manipulés avec précaution. Les femmes enceintes doivent être averties et éviter la manipulation des cytotoxiques. Tout contenant cassé doit être traité avec les mêmes précautions et considéré comme un déchet contaminé. L'élimination des déchets contaminés se fait par incinération dans des conteneurs rigides étiquetés à cet effet.

Ces dispositions peuvent être envisagées dans le cadre du réseau de cancérologie (circulaire DGS/DH/98, n° 98/188, du 24 mars 1998) en collaboration avec toute structure adaptée et remplissant les conditions requises.

DC/CONTRE-INDICATIONS

- Hypersensibilité connue à la gemcitabine.
- Association concomitante de la gemcitabine et de la radiothérapie, en raison du risque de radiosensibilisation, d'apparition de fibroses pulmonaires et œsophagiennes sévères.

- Association gemcitabine/cisplatine chez l'insuffisant rénal sévère.

DC/MISES EN GARDE et PRÉCAUTIONS D'EMPLOI

Mises en garde :

En cas d'utilisation successive, la possibilité de radiosensibilisation grave justifie qu'un intervalle d'au moins 4 semaines sépare la chimiothérapie par la gemcitabine de la radiothérapie. Ce délai peut être raccourci si l'état clinique du patient l'exige.
Une toxicité accrue a été démontrée en cas d'allongement du temps de perfusion et d'augmentation de la fréquence d'administration.
Comme d'autres agents cytotoxiques, la gemcitabine peut induire une aplasie médullaire qui se traduit par une anémie, une leucopénie et une thrombocytopénie. Cette thrombocytopénie est souvent sévère et nécessite parfois le recours aux transfusions plaquettaires. Toutefois, la myélosuppression est de courte durée et ne nécessite généralement pas de réduction de la dose et rarement l'arrêt du traitement.
Hypersensibilité : de rares cas de réaction anaphylactique ont été rapportés.

Précautions d'emploi :

Les patients recevant de la gemcitabine doivent faire l'objet d'un suivi attentif. Les paramètres biologiques doivent être contrôlés par un laboratoire d'analyses médicales. Le traitement de l'éventuelle toxicité du médicament peut être requis.
Le traitement doit être instauré avec prudence chez les patients dont la fonction médullaire est déficiente. Comme pour d'autres cytolytiques, il convient d'envisager la possibilité d'aplasie médullaire cumulée en cas de chimiothérapie combinée ou séquentielle.
Les patients recevant de la gemcitabine doivent faire l'objet avant chaque administration d'un suivi hématologique avec numération formule sanguine et plaquettes. Une suspension ou une modification du traitement doit être envisagée chaque fois qu'une toxicité médullaire induite par le médicament est décelée (cf Posologie et Mode d'administration). Les numérations globulaires peuvent continuer à diminuer après l'arrêt du traitement.
La gemcitabine sera utilisée avec prudence chez les insuffisants hépatiques en l'absence d'étude.
Une insuffisance rénale avec une clairance de la créatinine comprise entre 30 ml/min et 80 ml/min n'a pas d'effet significatif sur la pharmacocinétique de la gemcitabine.
L'utilisation de la gemcitabine devra être évitée chez la femme enceinte ou qui allaite (cf Grossesse et Allaitement).

DC/GROSSESSE et ALLAITEMENT

L'innocuité de la gemcitabine chez la femme enceinte n'a pas été établie. Le médicament s'étant révélé embryotoxique, fœtotoxique et tératogène dans les expérimentations animales, l'utilisation de la gemcitabine doit être évitée au cours de la grossesse et de l'allaitement à cause du risque potentiel pour le fœtus et l'enfant.

DC/CONDUITE et UTILISATION DE MACHINES

La gemcitabine pouvant induire une somnolence, les patients doivent s'abstenir de conduire un véhicule ou d'utiliser des machines tant que ce type de réaction n'a pas été exclu.

DC/EFFETS INDÉSIRABLES

Hématologiques :
La gemcitabine peut induire une aplasie médullaire, entraînant une anémie, une leucopénie et une thrombocytopénie. La myélosuppression est généralement modérée, elle est plus prononcée pour la lignée granulocytaire. La thrombocytémie est un autre effet fréquemment rapporté.
Hépatiques :
Des augmentations des transaminases hépatiques sont observées. Elles sont habituellement faibles, transitoires et ne nécessitent que rarement l'arrêt du traitement. La prudence s'impose toutefois chez les patients dont la fonction hépatique est altérée.
Œso-gastro-intestinaux :
Nausées, parfois accompagnées de vomissements. Ces effets secondaires justifient des mesures thérapeutiques dans approximativement 20 % des cas, mais n'imposent que rarement la diminution de la dose et sont faciles à traiter par les antiémétiques classiques. Diarrhées, toxicité buccale à type de mucite.
Pulmonaires :
Dans les heures qui suivent l'injection de gemcitabine, les patients peuvent présenter une

dyspnée, qui est généralement d'intensité faible et de courte durée. Elle nécessite rarement une réduction de la posologie et disparaît habituellement sans traitement spécifique. Son mécanisme est inconnu et sa relation avec la gemcitabine n'est pas claire.
Des cas d'œdème pulmonaire, de pneumopathies interstitielles et de syndrome de détresse respiratoire de l'adulte (ARDS), d'étiologie inconnue, ont été rapportés au cours du traitement par gemcitabine. Dès leur survenue, l'arrêt de la gemcitabine doit être envisagé.

Rénaux :

Une protéinurie et une hématurie modérées surviennent chez près de la moitié des patients, mais sont rarement significatives sur le plan clinique ; elles ne sont habituellement pas associées à des modifications de la créatinine sérique ou de l'urémie. On a cependant rapporté quelques cas d'insuffisance rénale d'étiologie incertaine. Aucune toxicité rénale cumulative n'a été observée (cf Mises en garde et Précautions d'emploi).
Des manifestations cliniques compatibles avec un syndrome hémolytique et urémique ont été rapportées chez les patients recevant de la gemcitabine. Le traitement par gemcitabine doit être interrompu dès les premiers signes d'anémie hémolytique micro-angiopathique tels qu'une chute brutale de l'hémoglobine avec thrombocytopénie concomitante, élévation de la bilirubine sérique, de la créatinine sérique, de l'urée sanguine ou de la LDH. L'insuffisance rénale peut ne pas être réversible, même à l'arrêt du traitement, et une dialyse peut être nécessaire.

Allergiques :

Des éruptions peuvent survenir et s'accompagner de prurit. L'éruption est habituellement faible, ne nécessite pas de réduction posologique et répond à un traitement local. Une desquamation, une vésiculation et une ulcération sont des effets secondaires rapportés occasionnellement.
Un bronchospasme a parfois été rapporté. Ce bronchospasme est habituellement d'intensité modérée et passager, mais il peut requérir un traitement parentéral. La gemcitabine ne doit pas être administrée aux patients ayant une hypersensibilité connue à ce produit. De rares cas de réaction anaphylactique ont été rapportés.

Cardiaques :

Des cas d'infarctus du myocarde, d'insuffisance cardiaque congestive et d'arythmie ont été observés.
On a rapporté quelques cas d'hypotension.

Cutanés :

Des manifestations cutanéomusculaires sévères à type de dermatopolymyosite, au niveau du site antérieurement irradié, ont été rapportées après administration successive de radiothérapie et de gemcitabine.

Autres :

Un syndrome grippal rarement sévère peut survenir. Il est généralement de courte durée et nécessite rarement une diminution de la posologie. Fièvre, céphalées, dorsalgie, frissons, myalgies, asthénie et anorexie sont les symptômes les plus communément rapportés. De même, une toux, une rhinite, des malaises, des sueurs et une insomnie sont couramment signalés. La fièvre et l'asthénie sont également rapportées comme symptômes isolés. Le mécanisme à la base de cette toxicité est inconnu. Le paracétamol peut en atténuer les symptômes.
Œdème périphérique, très rarement œdème facial. L'œdème périphérique est habituellement modéré et n'impose que rarement une réduction de la posologie, mais peut être douloureux ; il est généralement réversible après l'arrêt de la gemcitabine. Le mécanisme à la base de cette toxicité est inconnu. Il n'y a aucune association avec des signes d'insuffisance cardiaque, hépatique ou rénale.
Les effets secondaires suivants sont aussi couramment rapportés : alopécie (en général minime), somnolence.

DC/SURDOSAGE

Il n'y a pas d'antidote connu à la gemcitabine. Des doses uniques allant jusqu'à 5,7 g/m^2 ont fait l'objet de perfusion IV en 30 minutes toutes les deux semaines avec une toxicité acceptable sur le plan clinique. Si on suspecte un surdosage, le patient fera l'objet d'un suivi comprenant les numérations globulaires appropriées et recevra si nécessaire un traitement d'appoint.

PP/PHARMACODYNAMIE

Antimétabolite (L : anticancéreux, immunosuppresseurs).

Activité cytotoxique in vitro :

La gemcitabine possède une activité cytotoxique significative sur diverses cellules murines et cellules tumorales humaines en culture. La gemcitabine est un antimétabolite spécifique de la phase S du cycle cellulaire (phase de synthèse de l'ADN), elle bloque dans certaines circonstances la progression cellulaire au-delà de la phase G1/S. In vitro, l'action cytotoxique de la gemcitabine dépend à la fois de sa concentration et du temps.

Activité antitumorale préclinique :

Dans les modèles de tumeurs chez l'animal, l'activité antitumorale de la gemcitabine dépend du schéma d'administration. Administrée quotidiennement, la gemcitabine entraîne la mort des animaux avec une activité antitumorale minimale. Toutefois, lorsque l'on a recours à un schéma thérapeutique avec administration tous les trois ou quatre jours, la gemcitabine peut être administrée à des doses non létales pourvues d'une excellente action antitumorale sur un grand nombre de tumeurs de la souris.

Métabolisme cellulaire et mécanismes d'action :

La gemcitabine (dFdC) est métabolisée dans les cellules par des nucléosides kinases en nucléosides diphosphate (dFdCDP) et triphosphate (dFdCTP) actifs. L'action cytotoxique de la gemcitabine semble due à l'inhibition de la synthèse de l'ADN par la double action du dFdCDP et du dFdCTP. D'abord, le dFdCDP inhibe la ribonucléotide réductase qui agit comme unique catalyseur des réactions qui produisent des désoxynucléosides triphosphates destinés à la synthèse de l'ADN. L'inhibition de cette enzyme par le dFdCDP entraîne une réduction des concentrations de désoxynucléosides en général et du dCTP en particulier. En second lieu, le dFdCTP entre en compétition avec le dCTP pour son incorporation dans l'ADN (autopotentialisation). De la même manière, une faible quantité de gemcitabine peut aussi être incorporée dans l'ARN. Ainsi, la réduction de la concentration intracellulaire du dCTP potentialise l'incorporation du dFdCTP dans l'ADN. L'ADN polymérase epsilon est incapable d'écarter la gemcitabine et de réparer les chaînes d'ADN en cours de formation. Après incorporation de la gemcitabine dans l'ADN, un nucléotide supplémentaire s'ajoute aux chaînes d'ADN en cours d'élongation. A la suite de cette adjonction, on assiste à une inhibition complète de la synthèse de l'ADN (terminaison de chaîne masquée). Après son incorporation dans l'ADN, la gemcitabine induit le processus de lyse cellulaire programmée, connu sous le nom d'apoptose.

PP/PHARMACOCINÉTIQUE

Pharmacocinétique de la gemcitabine :

Les pics plasmatiques mesurés immédiatement après l'administration d'une dose de 1 000 mg/m^2 en perfusion de 30 minutes varient de 10 à 40 µg/ml. La demi-vie moyenne terminale est de 17 minutes (extrêmes : 11 et 26 min). Le volume de distribution moyen du compartiment central est de 11 l/m^2 (extrêmes : 5 et 21 l/m^2) et le volume de distribution moyen à l'état d'équilibre (Vss) de 17 l/m^2 (extrêmes : 9 et 30 l/m^2). La fixation aux protéines plasmatiques est négligeable et la distribution tissulaire non extensive. La clairance systémique moyenne est de 90 l/h/m^2 (extrêmes : 40 et 130 l/h/m^2). Chez la femme, la clairance est approximativement 30 % plus faible que chez l'homme. Néanmoins, aux doses recommandées, ceci ne requiert pas de diminution de la posologie. Moins de 10 % de la dose administrée sont excrétés sous forme inchangée dans les urines. La valeur de la clairance rénale moyenne est de 2-7 l/h/m^2.

Métabolisme :

La gemcitabine est rapidement métabolisée par la cytidine déaminase dans le foie, les reins, le sang et les autres tissus. Le métabolisme intracellulaire de la gemcitabine produit des mono, di et triphosphates de gemcitabine (dFdCMP, dFdCDP et dFdCTP) parmi lesquels les dFdCDP et dFdCTP sont considérés actifs. Ces métabolites intracellulaires n'ont pas été détectés dans le plasma et l'urine. Le métabolite principal, 2'-déoxy-2', 2'-difluorouridine (dFdU), présent dans le plasma et l'urine est quant à lui inactif.

- Cinétique du dFdCTP :

Ce métabolite se trouve dans les cellules mononucléaires circulantes. Les informations qui suivent ont trait à ces cellules.

La demi-vie d'élimination terminale varie de 0,7 à 12 heures. Les concentrations intracellulaires augmentent en fonction de la dose de gemcitabine : des doses comprises entre 35 et 350 mg/m^2/30 min donnent des concentrations à l'état d'équilibre de 0,4 à 5 µg/ml. Au-delà de concentrations plasmatiques de gemcitabine de 5 µg/ml, les taux de dFdCTP cessent d'augmenter, ce qui suggère que la formation est saturable dans ces cellules. Les concentrations plasmatiques de gemcitabine consécutives à une dose de 1 000 mg/m^2/30 min sont supérieures à 5 µg/ml

pendant près de 30 minutes après la fin de la perfusion et supérieures à 0,4 µg/ml pendant l'heure qui suit.
- Cinétique du dFdU :
Le pic plasmatique, 3 à 15 minutes après la fin d'une perfusion de 1 000 mg/m^2 en 30 minutes, varie de 28 à 52 µg/ml. Les concentrations les plus basses après une administration hebdomadaire s'échelonnent de 0,07 à 1,12 µg/ml, sans accumulation apparente. Les concentrations plasmatiques diminuent selon une courbe triphasique. La demi-vie moyenne de la phase terminale est de 65 heures (extrêmes : 33 et 84 heures) ; le dFdU formé représente 91 à 98 % de la clairance de la gemcitabine. Le volume de distribution moyen du compartiment central est de 18 l/m^2 (extrêmes : 11 et 22 l/m^2) et le volume de distribution moyen à l'état d'équilibre (Vss) de 150 l/m^2 (extrêmes : 96 et 228 l/m^2). La distribution tissulaire est importante et la clairance moyenne apparente représentent 2,5 l/h/m^2 (extrêmes : 1 et 4 l/h/m^2). L'élimination se fait par voie urinaire.
- Élimination globale :
Pendant la semaine qui suit l'administration, 92 à 98 % de la dose de gemcitabine administrée sont retrouvés, 99 % dans les urines, essentiellement sous forme de dFdU ; 1 % s'élimine par voie fécale.

PP/SÉCURITÉ PRÉCLINIQUE

Lors d'études à doses répétées pouvant atteindre 6 mois, réalisées chez la souris et le chien, l'observation principale a été la suppression de l'hématopoïèse. Ces effets, liés aux propriétés cytotoxiques de la substance, étaient réversibles après l'arrêt du traitement. La sévérité de l'effet dépendait du schéma thérapeutique et de la dose.
Oncogénicité, mutagénicité, fertilité :
Des altérations cytogénétiques ont été provoquées par la gemcitabine lors d'un test in vivo. La gemcitabine a provoqué des mutations génétiques lors d'un test in vitro sur une lignée de cellules de lymphome de souris (L5178Y).
La gemcitabine entraîne chez les souris mâles une hypospermatogenèse réversible, dépendant du schéma thérapeutique et de la dose. Bien que les études animales aient montré un effet de la gemcitabine sur la fertilité masculine, cet effet n'a pas été observé sur la fertilité féminine.

DP/INCOMPATIBILITÉS

Bien qu'aucune incompatibilité n'ait été démontrée, il est néanmoins recommandé de ne pas mélanger les solutions de gemcitabine avec d'autres médicaments.

DP/CONDITIONS PARTICULIÈRES DE CONSERVATION

A conserver à une température ne dépassant pas 30 °C.
Après reconstitution : les solutions de gemcitabine seront conservées à une température ne dépassant pas 30 °C et utilisées dans les 24 heures. Rejeter la solution non utilisée.
La réfrigération est déconseillée car elle entraîne une cristallisation.

DP/MODALITÉS DE MANIPULATION

La manipulation de ce cytotoxique par le personnel infirmier ou médical nécessite un ensemble de précautions permettant d'assurer la protection du manipulateur et de son environnement (cf Posologie et Mode d'administration).
Les précautions usuelles de manipulation des médicaments anticancéreux doivent être respectées. Le seul diluant recommandé pour reconstituer la poudre stérile de gemcitabine est le chlorure de sodium à 0,9 % pour préparations injectables sans conservateur. Bien qu'aucune incompatibilité n'ait été démontrée, il est néanmoins recommandé de ne pas mélanger les solutions de gemcitabine avec d'autres médicaments. Pour des raisons de solubilité, la limite supérieure de concentration de la gemcitabine après reconstitution est de 40 mg/ml. La reconstitution à des concentrations supérieures à 40 mg/ml est à éviter en raison d'une dissolution incomplète.
Reconstitution : ajouter au moins 5 ml de chlorure de sodium pour préparations injectables à 0,9 % au flacon à 200 mg ou au moins 25 ml de chlorure de sodium à 0,9 % pour préparations injectables au flacon à 1 000 mg. Agiter jusqu'à dissolution. Les solutions de gemcitabine peuvent être administrées comme préparées ci-dessus ou être diluées avec du chlorure de sodium pour préparations injectables à 0,9 %.
Avant d'être administrées, les substances pour usage parentéral doivent faire l'objet d'une inspection visuelle pour détecter la présence éventuelle de particules ou d'une décoloration. Comme tout cytostatique, le chlorhydrate de gemcitabine doit être manipulé avec prudence.

Les produits non utilisés doivent être détruits conformément aux procédures hospitalières de traitement des déchets cytotoxiques.

LISTE I

A 559 674.7 (1996, rév 02.10.2000) lyoph à 200 mg.
MM
559 675.3 (1996, rév 02.10.2000) lyoph à 1 000 mg.
Réservé à l'usage hospitalier. Collect.

LILLY FRANCE SA
203, bureaux de la Colline. 92213 Saint-Cloud
Tél : 01 49 11 34 34
Information médicale : Tél (n° Vert) : 08 00 00 36 36
Pharmacovigilance : Tél (n° Vert) : 08 00 39 46 34
Site web : http://www.lilly.fr

ANNEXE 2 : NOTE D'INFORMATION ET DE CONSENTEMENT ECLAIRE POUR L'ETUDE CLINIQUE DESTINE AU PATIENT

Etude FFCD 1004 « PHARMACOGENETIQUE DE LA GEMCITABINE: ETUDE DE L'IMPACT DU POLYMORPHISME GENETIQUE DE LA CYTIDINE DEAMINASE (CDA) SUR LA TOXICITE DANS LES ADENOCARCINOMES PANCREATIQUES RESEQUES»
EudraCT N° : 2010-022987-11

Promoteur de l'essai : Fédération Francophone de Cancérologie Digestive (FFCD), Faculté de Médecine, 7 Boulevard Jeanne d'Arc, BP 87900, 21079 Dijon Cedex, France

Madame, Monsieur,

Le Docteur, Nom/ Prénom…………………………………………
Adresse et n° de tél ………………………………………………………………, médecin investigateur, vous propose de participer à cette recherche biomédicale.
Afin d'éclairer votre décision, il vous demande de prendre connaissance des informations suivantes :

BUT ET JUTIFICATION DE L'ETUDE

La prise en charge thérapeutique de votre pathologie fait appel à une chimiothérapie anticancéreuse, la gemcitabine (Gemzar®). Comme tout autre médicament, ce médicament peut provoquer des effets indésirables, notamment chez certains patients prédisposés.

Cette étude est réalisée pour permettre de mieux comprendre ces mécanismes de prédisposition et ceci afin d'améliorer dans l'avenir la prise en charge thérapeutique des futurs patients qui seront appelés à recevoir de la gemcitabine. L'étude évaluera les concentrations de ce médicament dans le sang et l'activité d'une enzyme, la cytidine déaminase (CDA), responsable de sa dégradation. Finalement, l'étude permettra d'évaluer la capacité du CDA à prédire la survenue d'une toxicité hématologique sévère.

Des prélèvements sanguins seront réalisés; afin d'une part d'évaluer l'activité de l'enzyme CDA et de rechercher d'éventuelles mutations s'y rapportant. Il s'agit de mutations génétiques affectant le gène codant pour la CDA et pouvant modifier son activité. Ces mutations n'ont aucune influence sur votre vie quotidienne mais peuvent modifier les concentrations du médicament dans le sang. Votre statut CDA sera évalué avant le début du traitement. D'autre part, 4 prélèvements successifs (optionnels) permettront de doser la gemcitabine dans le sang à différents moments (étude de pharmacocinétique).

DEROULEMENT DE L'ETUDE

120 patients participeront à cette étude dans différents centres français.

Votre suivi sera identique à celui de tout patient dans la même situation (même hors protocole), il sera d'au moins un an après le dernier patient inclus dans l'étude..

La consultation médicale d'inclusion prévue par le protocole de l'étude correspond à une consultation habituelle de votre suivi.

L'étude comportera :

- 1 prélèvement sanguin (2 tubes : l'un de 10ml, l'autre de 5ml) réalisé avant le début du traitement par gemcitabine (soit dans les 8 semaines post-opératoires). Elle comporte 6 cycles de 4 semaines (perfusion intraveineuses hebdomadaires) selon les recommandations. Ces échantillons sanguins serviront à évaluer l'activité de l'enzyme

CDA et à rechercher une éventuelle anomalie pouvant induire son mauvais fonctionnement.

- de façon optionnelle : 4 prélèvements sanguins successifs (4 tubes de 5ml), au début et à la fin de la première perfusion de gemcitabine, puis 90 minutes et 120 minutes après. Un cathéter intra-veineux vous sera placé au début de la perfusion au niveau du pli du coude. Tous les prélèvements se feront à partir de ce même cathéter : il n'y aura donc qu'une seule piqûre Ces échantillons serviront au dosage du taux sanguin de gemcitabine.

Les échantillons ainsi que les résultats de ces analyses seront strictement anonymes.

On ne peut exclure que les résultats obtenus par l'utilisation de matériels biologiques puissent conduire à l'obtention de droits exclusifs reposant sur des découvertes liées à la recherche. Vous ne recevrez aucune contre partie financière. Au cas où la FFCD, promoteur de l'étude, bénéficierait d'un financement, il serait réinvesti dans la recherche contre le cancer dans le seul but d'en améliorer le traitement.

EFFETS INDESIRABLES EVENTUELS LIES AU TRAITEMENT

L'efficacité et la tolérance de ce médicament sont connues et sa commercialisation et son utilisation autorisées en France depuis plusieurs années

Votre tolérance hématologique (globules rouges, globules blancs, plaquettes) au médicament sera évaluée 7 jours après l'administration de la gemcitabine. En effet, l'un des principaux effets indésirables de ce médicament est la diminution possible des taux sanguins de globules blancs et des plaquettes.

Cet effet est inconstant et lorsqu'il survient il est le plus souvent sans conséquence. Dans de rares cas, il peut vous exposer à un risque d'infection ou d'hémorragie. De ce fait entre deux cycles de traitement, en cas d'hémorragie ou en cas de fièvre inexpliquée supérieure a 38°, il faudra effectuer une prise de sang pour numération formule sanguine et plaquettes et prévenir d'urgence votre médecin

Les autres toxicités éventuelles seront également surveillées lors d'une consultation avec le médecin investigateur. Les toxicités reconnues de ce médicament sont principalement, outre la toxicité hématologique, une protéinurie, une toxicité digestive transitoire et d'intensité modérée (nausées, vomissement, troubles du transit, muscites), pour laquelle un traitement pourra être prescrit afin d'en limiter les conséquences.

BENEFICES ATTENDUS ET CONTRAINTES

Cette étude a pour objectif de mieux comprendre les mécanismes de prédisposition aux effets indésirables liés à la gemcitabine et ceci afin d'améliorer la prise en charge thérapeutique des patients appelés à recevoir de la gemcitabine à l'avenir.

Excepté les prélèvements supplémentaires décrits ci-dessus, il n'y a pas de contraintes particulières liées à cette étude, comparées à celle du traitement et du suivi habituel de votre maladie.

PROTECTION DES PATIENTS ET CONFIDENTIALITE

Vous avez le droit de refuser de participer ou de vous retirer de l'étude à n'importe quel moment et sans donner la moindre raison, sans encourir aucune responsabilité ni aucun préjudice de ce fait. Cela ne changera pas vos relations avec les médecins ni les soins que vous recevrez et ceci sans donner la moindre raison.

Si vous participez à cette étude, vous ne pourrez pas participer à une autre recherche, étudiant un autre traitement que la gemcitabine et ce pour des raisons d'interactions médicamenteuses potentielles.

Cette recherche a reçu l'avis favorable du Comité de Protection des Personnes (CPP) Sud Méditerranée I le 09/03/2011 et l'autorisation de l'AFSSAPS le 31/01/2011.

Votre participation à ce projet de recherche biomédicale est couverte par une police d'assurance souscrite par le promoteur de l'étude auprès de la compagnie d'assurance HDI-GERLING, n° (1680) 90712, selon les termes de l'article L1121-10 du code de la santé publique.

Pour participer à cet essai, vous devrez bénéficier d'un régime d'assurance maladie.

Les informations médicales collectées durant cette étude seront enregistrées anonymement et confidentiellement ; ces informations peuvent être consultées par les représentants du promoteur ou par les Autorités Sanitaires Françaises.

En application de l'article 54 (alinéa 5) de la loi 78-17 du 6 janvier 1978, amendé par la loi du 9 août 2004, la FFCD a fait une déclaration simplifiée (méthodologie de référence MR01, méthodologie de référence pour les traitements de données personnelles opérés dans le cadre des recherches biomédicales) auprès de la Commission Nationale Informatique et Libertés (CNIL) pour tous ses fichiers informatiques. Vous pouvez à tout moment exercer votre droit d'accès et de rectification par le biais du médecin responsable de cette étude (article 40 de la loi de 2004).

Vous avez le droit de recevoir toute information concernant votre santé détenue par l'investigateur au cours ou à l'issue de la recherche, ainsi que de toute autre nouvelle donnée concernant l'étude, qui pourrait modifier votre décision de poursuivre votre participation.

À l'issue du projet de recherche et si vous le souhaitez, le médecin investigateur pourra vous informer des résultats globaux de ces recherches une fois qu'ils seront disponibles, comme le prévoit l'article L.1122-1 de la loi N° 2002-303 du 4 mars 2002.

CONSENTEMENT ECLAIRE

Essai FFCD 1004 « PHARMACOGENETIQUE DE LA GEMCITABINE: ETUDE DE L'IMPACT DU POLYMORPHISME GENETIQUE DE LA CYTIDINE DEAMINASE (CDA) SUR LA TOXICITE DANS LES ADENOCARCINOMES PANCREATIQUES RESEQUES»

EudraCT N° : 2010-022987-11

Promoteur de l'essai : Fédération Francophone de Cancérologie Digestive (FFCD), Faculté de Médecine, 7 Boulevard Jeanne d'Arc, BP 87900, 21079 Dijon Cedex, France

Le Docteur, Nom/ Prénom…………………………………………

Adresse et n° de tél………………………………………………………………………………,
médecin investigateur, m'a proposé de participer à cette recherche biomédicale.

Je déclare :

- avoir reçu la lettre d'information de cette étude, avoir disposé d'un temps de réflexion me permettant en particulier d'en discuter avec ma famille, mes proches, et/ou mon médecin traitant, avant de donner mon consentement,
- avoir pu poser toutes les questions que je voulais et avoir reçu les réponses adaptées
- savoir que le Comité de Protection des Personnes Sud Méditerrané I a été consulté et a donné un avis favorable pour cette étude,
- savoir que j'ai la possibilité de retirer mon consentement à tout moment, sans avoir à me justifier et sans entraîner en aucune manière un changement dans la qualité des soins qui me seront donnés,
- savoir que les informations me concernant resteront strictement confidentielles et seront informatisées de façon anonyme,
- savoir que je bénéficie, à tout moment, du droit d'accès et de rectification des informations me concernant (Loi n°78-17 du 6 janvier 1978 relative à l'informatique, aux fichiers et aux libertés, modifiée par la loi n°94-548 du 1er juillet 1994, du 04 mars 2002 et du mois d'août 2004).

Je peux à tout moment demander toute information complémentaire au Docteur investigateur ci-dessus ou en cas d'urgence à un membre de son équipe.

J'accepte :

☐ le prélèvement réalisé avant le traitement permettant de mesurer l'activité de mon enzyme CDA

☐ les prélèvements le jour de ma première cure de chimiothérapie mesurant les taux sanguins de gemcitabine dans mon sang

En cas de collaboration de la FFCD avec un tiers j'accepte que mes matériels biologiques soient utilisés par ce tiers (autre institution/organisme universitaire ou compagnie pharmaceutique). Les prélèvements sanguins nécessaires à l'évaluation de l'activité CDA et le cas échéant aux dosages de médicament seront détruits après analyse.

Les personnes chargées du contrôle qualité de cette étude et dûment mandatées à cet effet par le promoteur auront accès à mes données individuelles strictement nécessaires à ce contrôle ; ces personnes sont soumises au secret professionnel (article L1121-3 du code de la santé publique).

Fait à .... le ....

L'investigateur (Nom, prénom):	Le patient (Nom, prénom):
Signature :	Signature :

Ce document est à réaliser en 2 exemplaires originaux, dont l'un doit être gardé 15 ans par l'investigateur et l'autre remis à la personne donnant son consentement.

Annexe 3 : CRITERES DE TOXICITE NCI-CTC version 4.0

Se référer à l'échelle d'évaluation de la toxicité CTCAE version 4.0 traduite en française par la FFCD, jointe séparément (fascicule bleu).

La version originale anglaise est téléchargeable sur le site du NCI

http://ctep.cancer.gov/

Common Terminology Criteria for Adverse Events v4.0 (CTCAE)
(Publish Date May 28, 2009)

ANNEXE 4 : FICHES DE PRELEVEMENT

FFCD 1004 - PHARMACOGENETIQUE DE LA GEMCITABINE : ETUDE DE L'IMPACT DU POLYMORPHISME GENETIQUE DE LA CYTIDINE DEAMINASE (CDA) SUR LA TOXICITE DANS LES ADENOCARCINOMES PANCREATIQUES RESEQUES

Fiche de PRELEVEMENT SANGUIN Typage CDA (étude PHENOTYPIQUE)

Destiné au Laboratoire de Transfert d'Oncologie Biologique

Faculté de Médecine – Secteur Nord – Bd Pierre Dramard – 13015 Marseille – tel 04.91.69.88.82

PROVENANCE

Centre :
Investigateur :

Tampon du service + Signature :

PATIENT

Initiales : nom/prénom

Code patient :

Résultat CRP =
Date du prélèvement sanguin :

Date de signature du consentement :

PRELEVEMENT – CENTRIFUGATION – STOCKAGE

1 tube sec sans gel séparateur (tube rouge), Centrifuger (20 min, 2500 rpm, 4°C), aliquoter dans des cryotubes identifiés avec le n° du patient de l'étude + initiales nom/prénom, congeler à -80°C jusqu'à envoi groupé sur carboglace en cours d'étude ou en fin d'étude

Date du prélèvement :

Nombre de cryotubes :

ENREGISTREMENT

Partie réservée au Laboratoire de Transfert en Oncologie Biologique

Date de réception :

Heure de réception :

Réfrigéré : ☐ oui ☐ non

N° d'enregistrement

STOCKAGE

Nombre de cryotubes de sérum :

Coller une étiquette d'identification du patient sur chaque tube

Commentaires :

Contact : **Dr Joseph CICCOLINI** – Laboratoire de Transfert – Médecine Nord 13015 Marseille joseph.ciccolini@univmed.fr

FFCD 1004 - PHARMACOGENETIQUE DE LA GEMCITABINE: ETUDE DE L'IMPACT DU POLYMORPHISME GENETIQUE DE LA CYTIDINE DEAMINASE (CDA) SUR LA TOXICITE DANS LES ADENOCARCINOMES PANCREATIQUES RESEQUES

Fiche de PRELEVEMENT SANGUIN Typage CDA (étude GENOTYPIQUE)

Destiné au Laboratoire de Transfert d'Oncologie Biologique
Faculté de Médecine – Secteur Nord – Bd Pierre Dramard – 13015 Marseille – tel 04.91.69.88.82

PROVENANCE

Centre :

Investigateur :

Tampon du service + Signature :

PATIENT

Initiales : nom/prénom

Code patient :

Résultat CRP :
Date du prélèvement :

Date de signature du consentement :

PRELEVEMENT

1 tube EDTA (tube mauve) – conserver à 4°C jusqu'au transfert en cryotube(s)(dans la journée au maximum) ; les cryotubes, identifiés avec le n° du patient de l'étude + initiales nom/prénom, sont stockés à -80°C, jusqu'à l'envoi groupé sur carboglace en cours et en fin d'étude

Date du prélèvement :

Nombre de cryotubes de sang total :

ENREGISTREMENT

Partie réservée au Laboratoire de Transfert en Oncologie Biologique

Date de réception :

Heure de réception :

Réfrigéré : ☐ oui ☐ non

N° d'enregistrement

STOCKAGE

Nombre de cryotubes de sang total :

Coller une étiquette d'identification du patient sur chaque tube

Commentaires :

Contact : **Joseph CICCOLINI** – Laboratoire de Transfert – Médecine Nord – joseph.ciccolini@univmed.fr

ANNEXE 5 : Règles de publication FFCD

REGLES DE PUBLICATION DE LA FFCD

La publication des essais de la FFCD dans un délai rapide et dans une revue de qualité est un objectif essentiel pour le développement des essais et le renom de la société. Cette publication doit être sous la responsabilité du Bureau qui décide du moment de la publication des résultats préliminaires d'une étude, et des résultats définitifs, et veille à ce que les délais soient tenus. Il désigne à cet effet un Comité de Rédaction (5 membres au maximum).

1) Le Comité de Rédaction comprend :

- Le coordinateur (ou les coordinateurs s'ils sont deux) qui a écrit le premier projet
- Eventuellement un spécialiste ayant fourni une contribution essentielle à l'analyse des données (biologiste)
- Le statisticien ayant effectué l'analyse des données
- Les contributeurs importants (inclusions > 10% des patients)

En cas d'essais coopératifs et dans la mesure où l'autre (ou les autres) association(s) a (ont) au moins 10 % de l'effectif, ce Comité est approuvé par la Commission d'Interface et comprend un représentant des autres associations à condition qu'il soit lui-même investigateur.

2) Le comité s'engage à publier dans un délai déterminé par le Bureau. Ce délai ne devra pas dépasser une année après la clôture d'un essai. S'il n'est pas en mesure de le faire, le Bureau désigne un rédacteur qui deviendra le premier auteur.

3) Les auteurs de la publication sont dans l'ordre en fonction du travail fourni et nombre de patients inclus :

- Le coordinateur ou le rédacteur principal
- Les membres du Comité de Rédaction de l'article ci-dessus
- Un nombre limité d'investigateurs (un par centre) ayant entré au moins 5 % des patients dans l'ordre de leur participation, et en règle un seul par centre mais pour certains centres le Comité peut décider deux investigateurs
- La FFCD est citée dans le titre après les auteurs. En cas d'essai coopératif, la première association citée est celle qui a initié l'essai et les autres sont mentionnées à condition qu'elles aient entré au moins 10 % des patients

Tous les participants sont cités en annexe. Le gestionnaire de l'étude (Technicien recherche clinique) est également cité. Il peut être cité dans les auteurs si le Bureau l'estime justifié.

Les partenaires sont remerciés.

Les auteurs et le promoteur reçoivent un manuscrit pour critiques avant l'envoi à une revue.

4) Communication orale à partir des résultats de l'essai

- Un investigateur peut, avec l'accord du Bureau et du coordinateur, présenter en son nom tout ou partie des résultats en communication orale. Les auteurs sont les mêmes que pour l'article écrit, mais l'ordre des auteurs pour les articles et les communications variera de façon équilibrée. Dans certains cas (études pathologiques, biologiques, écho-endoscopiques parallèles à un essai thérapeutique par exemple) d'autres auteurs pourront être choisis en fonction de leur travail. La FFCD et les autres associations, le cas échéant, doivent être citées.

ANNEXE 6: Certificat d'assurance

Bio... Courtier en assurances
Parc d'Innovation Bretagne sud
C.P. 142 - 56038 VANNES CEDEX
Tel. [illegible]
Fax [illegible]
biomail[illegible]

HDI GERLING

ATTESTATION D'ASSURANCE
RESPONSABILITE CIVILE
PROMOTEUR DE RECHERCHE BIOMEDICALE

ADHESION n° 201000176

Nous, soussignés HDI-GERLING - TOUR OPUS 12, 77, Esplanade de la Défense 92914 PARIS LA DEFENSE agissant en qualité d'assureur, attestons par la présente que :

FFCD
Féd. Francoph. de Cancérologie Digestive
Faculté de Médecine
7 Bvd Jeanne d'Arc
21079 DIJON CEDEX

a souscrit un contrat de Responsabilité Civile sous le n° (1680) 90712

Conforme aux dispositions légales et réglementaires Françaises sur les recherches biomédicales et notamment aux dispositions de la loi 88.1138 du 20/12/1988, modifiée par les textes subséquents: loi 90.86 du 23/01/1990, décret 91-440 du 14/05/1991, loi 94.630 du 25/07/1994, décret 97-888 du 01/10/1997, décret 2002-722 du 03/05/2002, loi 2004.806 du 09/08/2004, décret 2006-477 du 26/04/2006.

Description précise de la recherche assurée :

PHARMACOGENETIQUE DE LA GEMCITABINE : ETUDE DE L'IMPACT DU POLYMORPHISME GENETIQUE DE LA CYTIDINE DEAMINASE (CDA) SUR LA TOXICITE DANS LES ADENOCARCINOMES PANCREATIQUES RESEQUES
Protocole n° FFCD 1004

La garantie est conforme à l'obligation d'assurance instituée par les textes de la loi précitée, article L 1121-10 du Code de la Santé Publique, à la charge du promoteur, tant pour sa responsabilité que pour celle des intervenants.

La garantie prévue au contrat restera acquise à l'Assuré en cas de modification affectant la prise d'effet du protocole.

La présente attestation est valable pour la durée de la recherche concernée et sa présentation vaut présomption de garantie à la charge de l'assureur.

Fait , le 22 novembre 2010

Le Courtier
BIOMEDIC INSURE INDUSTRIES
Jean-Pierre DANIEL
Parc d'Innovation Bretagne sud
C.P. 142 - [illegible] CEDEX
Tel. [illegible]
Fax +33(0)2 [illegible]
biomail[illegible]

L'Assureur
HDI-GERLING
HDI-Gerling Industrie Versicherung AG
Capital [illegible] EUR
TOUR OPUS 12, LA DEFENSE 9
77, Esplanade du Général de Gaulle
F 92914 PARIS LA DEFENSE CEDEX
Tél. : +33 1 44 05 [illegible] - Fax +33 1 44 05 [illegible]

502/2010

ANNEXE 7: Avis du CPP Sud Méditerranée I

COMITE DE PROTECTION DES PERSONNES SUD MÉDITERRANÉE I

Président
Professeur Jean-Albert GASTAUT

AVIS

Collège technique

Personnes qualifiées en recherche

Jean-Albert GASTAUT
Geneviève MACQUART MOULIN
Laetitia HUIART(méthodologiste)
Yves JAMMES
Jacques ALBANESE
Jean-Louis ROMETTE
Jean GAUDART(méthodologiste)
Dominique GENRE

Médecins généralistes

Gérard SPANO

Pharmaciens hospitaliers

Bruno LACARELLE
Christine PENOT RAGON

Infirmières

Dominique CHANAUD
Lydie CAMOIN

Collège social

Personnes qualifiées en éthique

Christine ASSAÏANTE
Célia CHISCHPORTICH

Psychologues

Pierre DE ALCALA
Christian BOCCARDI

Travailleurs sociaux

Christine FAURE
Claire GIUDICELLI-PANZA

Juristes

Dominique GIOCANTI
Annagrazia ALTAVILLA
Andrée MILLIET
Guillaume CHEROUATI

Représentants d'associations et usagers

Bernard PANZA
Françoise MOULARD

Philippe HANRIAT

Le Comité de Protection des Personnes Sud-Méditerranée I, agréé par arrêté ministériel en date du 12 juin 2006, constitué selon l'arrêté du Préfet de la Région Provence Alpes Côte d'Azur en date du 5 février 2010,

en application du code de la santé publique et de la réglementation en vigueur relative aux recherches biomédicales portant sur un projet de recherche biomédicale portant sur un médicament à usage humain.

ayant été saisi par un courrier de la Fédération Francophone de Cancérologie Digestive, promoteur d'un dossier de recherche biomédicale intitulée :

« PHARMACOGENETIQUE DE LA GEMCITABINE : ETUDE DE L'IMPACT DU POLYMORPHISME GENETIQUE DE LA CYTIDINE DEAMINASE (CDA) SUR LA TOXICITE DANS LES ADENOCARCINOMES PANCREATIQUES RESEQUES»

Identifié sous le numéro ID RCB : 2010-022987-11 et dont l'investigateur (coordinateur) est Mme le Dr Laëtitia DAHAN

ayant, après vérification de la conformité réglementaire, enregistré ce dossier le **30 novembre 2010** sous la référence interne **10 62**,

Lors de sa séance plénière du **15 décembre 2010** au cours de laquelle
Mesdames A. ALTAVILLA, C. ASSAÏANTE, L. CAMOIN, D. GIOCANTI, L. HUIART, G. MACQUART MOULIN, F. MOULARD, C. PENOT RAGON,
Messieurs P. DE ALCALA, J-A GASTAUT, Y. JAMMES, G. SPANO

après avoir entendu le rapporteur du collège technique, le rapporteur du collège social et l'avis du méthodologiste ont délibéré,

Le Comité a demandé des modifications concernant le résumé, le protocole, la notice d'information et le formulaire de consentement

Le Comité, ayant reçu le 18 février 2011 les modifications demandées,

Lors de sa séance plénière du **9 mars 2011** au cours de laquelle :
Mesdames C. ASSAÏANTE, L. CAMOIN, C. CHISCHPORTICH, L. HUIART, G. MACQUART MOULIN, A. MILLIET, F. MOULARD
Messieurs C. BOCCARDI, J-A GASTAUT, Ph. HANRIAT, Y. JAMMES, B. LACARELLE

après avoir entendu le rapporteur du collège technique, le rapporteur du collège social et l'avis du méthodologiste ont délibéré,

Institut Paoli Calmettes
232 Bd Sainte-Marguerite - BP 156 - 13273 MARSEILLE
Tél. : 04.91.22.34.25 - Fax : 04.91.22.36.30 - E-mail : cppsudmed1@gmail.com
Site Internet : http://www.cppsudmediterranee1.fr

COMITE DE PROTECTION DES PERSONNES SUD MÉDITERRANÉE I

Président
Professeur Jean-Albert GASTAUT

Le Comité ayant examiné le dossier de recherche ainsi constitué :

- Le formulaire de demande d'autorisation daté et signé du 25 novembre 2010
- Résumé version n°1.1 du 9 février 2011
- Document additionnel daté et signé du 25 novembre 2010
- Protocole version n°1.1 du 9 février 2011
- Notice d'information version n°1.1 du 9 février 2011
- Formulaire de consentement version n°1.1 du 9 février 2011
- Liste des centres participants version n°1.1 du 10 février 2011
- CVs des investigateurs

Le Comité a émis un

AVIS FAVORABLE

à la mise en œuvre de cette recherche biomédicale, considérant que les conditions de validité de la recherche, notamment celles définies dans l'article L. 1123-7 du code de la santé publique, étaient réunies.

Professeur Jean-Albert GASTAUT

Institut Paoli Calmettes
232 Bd Sainte-Marguerite - BP 156 - 13273 MARSEILLE
Tél. : 04.91.22.34.25 - Fax : 04.91.22.36.30 - E-mail : cppsudmed1@gmail.com
Site Internet : http://www.cppsudmediterranee1.fr

ANNEXE 8 : Autorisation de l'AFSSAPS

Fax émis par : 33155873642 AFSSAPS U.E.C. 02-02-11 18:28 Pg: 1/1

afssaps
Agence française de sécurité sanitaire des produits de santé

AUTORISATION D'ESSAI CLINIQUE DE MEDICAMENT A USAGE HUMAIN

Nombre de pages : 1
(incluant la page de garde)

Envoi par Télécopie

Date : 3 1 JAN. 2011

**Identifiants de l'essai clinique**					
Titre	PHARMACOGENETIQUE DE LA GEMCITABINE : ETUDE DE L'IMPACT DU POLYMORPHISME GENETIQUE DE LA CYTIDINE DEAMINASE (CDA) SUR LA TOXICITE ET L'EFFICACITE THERAPEUTIQUE DANS LES ADENOCARCINOMES PANCREATIQUES RESEQUES				
Promoteur	Fédération Francophone de Cancérologie Digestive (FFCD)			Réf. CPP	Non disponible
Réf. Promoteur	FFCD 1004 - PRODIGE	N° EudraCT	2010-022987-11	Réf. Afssaps	A101374-31

**Expéditeur**	**Destinataire** (demandeur : nom / société / tél.)
AFSSAPS / DEMEB / Département de l'évaluation des essais cliniques et des médicaments à statut particulier<br>**Unité essais cliniques médicaments et hors produits de santé**	Martine SCHNEIDER<br>Fédération Francophone de CancérologieDigestive<br>03 80 39 34 83
Dossier suivi par : Gaëlle GUYADER<br>Tél : 33 (0) 1 55 87 36 41 / Fax : 33 (0) 1 55 87 36 42	Fax 03 80 38 18 41

**CPP** destinataire en copie	**Sud-Méditerranée I (Marseille 1)**	Fax	**04.91.74.56.16**	Code 12

Vu le code de la santé publique et notamment ses articles L. 1123-8, L. 1123-12 et vu le dossier de demande d'autorisation d'essai clinique adressé à l'Agence française de sécurité sanitaire des produits de santé (Afssaps) ;

L'autorisation mentionnée à l'article L. 1123-8 du code de la santé publique est accordée pour l'essai clinique cité en objet. Cette autorisation est valable pour toute la durée de l'essai à compter de la date de la présente décision.

Toutefois, conformément à l'article R. 1123-33 du code de la santé publique, la présente autorisation devient caduque si la recherche n'a pas débuté dans un délai d'un an.

Pour le Directeur Général et par délégation
Dr C. BELORGEY-BISMUT
Chef du Département de l'évaluation des Essais Cliniques et des Médicaments à Statut Particulier

3 1 JAN. 2011

Pour toute demande d'informations complémentaires concernant cette autorisation, je vous recommande de solliciter un rendez-vous téléphonique en adressant à cet effet un courriel uniquement sur la boîte **contact.suivi-ec@afssaps.sante.fr**. Je vous demande alors de veiller à reporter dans l'objet du message uniquement les mentions suivantes : **AEC/A101374-31**.

Par ailleurs, afin d'optimiser la gestion des dossiers de modifications substantielles (MS) que vous pourriez être amené(e) à déposer pour l'essai sus-cité, je vous recommande de les transmettre par courriel adressé uniquement sur la boîte **ams@afssaps.sante.fr**. Je vous précise qu'il vous est possible d'utiliser à cet effet le système de messagerie électronique sécurisée Eudralink. Lors de l'envoi de ces dossiers, je vous demande de veiller à reporter dans l'objet du message les mentions suivantes :

- pour les MS transmises à l'Afssaps pour information : **MSI/A101374-31**
- pour les MS soumises pour autorisation ou pour les dossiers mixtes (comportant des modifications soumises pour autorisation et d'autres pour information) : **MSA/A101374-31**

Si vous ne recevez pas toutes les pages de cette télécopie, veuillez contacter le secrétariat de Unité essais cliniques médicaments et hors produits de santé au : 33 (0) 1 55 87 36 41.

Confidentialité
Cette transmission est à l'attention exclusive du(des) destinataires ci-dessus mentionné(s) et peut contenir des informations privilégiées et/ou confidentielles. Si vous n'êtes pas le destinataire voulu ou une personne mandatée pour lui remettre cette transmission, vous avez reçu ce document par erreur et toute utilisation, révélation, copie ou communication de son contenu est interdite. Si vous avez reçu cette transmission par erreur, veuillez nous en informer par téléphone immédiatement et nous retourner le message original par courrier. Merci.

Confidentiality
This transmission is intended to the addressee(s) listed above only and may contain preferential or/and confidential information. If you are not the intended recipient, you are hereby notified that you have received this document by mistake and that any use, disclosure, copying or communication of the content of this transmission is prohibited. If you have received this transmission by mistake, please call us immediately and return the original message by mail. Thank you.

143/147, bd Anatole France - F-93285 Saint-Denis cedex - tél. +33 (0)1 55 87 30 00 - www.afssaps.sante.fr [illegible]
